# An *hcp3-vgrG3* intergenic region participates in EHEC T6SS expression in addition to the bidirectional promoter and H-NS

**DOI:** 10.1128/spectrum.03548-25

**Published:** 2026-04-14

**Authors:** Jaime Vazquez-Lopez, Landy Zambrano-Arguello, Juan M. Jimenez-Antaño, Luis A. Constantino-Jonapa, Jazmin Huerta-Cantillo, Fernando Navarro-Garcia

**Affiliations:** 1 Department of Cell Biology, Centro de Investigación y de Estudios Avanzados (Cinvestav) 42576 , Mexico City, Mexico; Universidad Andres Bello, Santiago, Chile

**Keywords:** type VI secretion system (T6SS), type III secretion system (T3SS), enterohemorrhagic *Escherichia coli *EHEC, Shiga toxin-producing *Escherichia coli *(STEC), RNA-binding proteins, bacterial competition, promoter regulation, H-NS, post-transcriptional regulation, transcriptional termination, promoter-swapping

## Abstract

Enterohemorrhagic *Escherichia coli* (EHEC) is notable for the Shiga-like toxin and several secretion systems and virulence factors that facilitate intestinal colonization and pathogenesis. EHEC low infectious dose may be in part due to its Type VI secretion system (T6SS), which could have antibacterial activity and perhaps damage host cells, although its function and mechanism are unknown. The aim of this study was to understand T6SS activation in EHEC by replacing its unknown native promoter with two inducible promoters. We found that the structural components encoded in the *tssB* operon are expressed as part of a single polycistronic operon. Excitingly, in the other direction, the *hcp3* region is formed of at least two separate operons, leading to incomplete T6SS activation and no secretion of T6SS substrates. Analysis of the region downstream of *hcp3* revealed a transcriptional terminator, a promoter region, and multiple transcription factor binding sites; thereby, we designated it as h3R. This regulatory sequence represses the translation of *vgrG3* but also contains a regulatory element that stabilizes the mRNA of *hcp3*, the gene located immediately upstream of *vgrG3*. Meanwhile, deletion of *hns* elevated T6SS mRNAs but was not sufficient for protein expression until further activated with DMEM or MM9, although not all proteins were expressed and protein secretion by other systems was modified. These data highlight the stringent regulatory control of the T6SS in EHEC and identify novel promoter regions that warrant further investigation to elucidate the role of this secretion system in bacterial competition and pathogenesis.

**IMPORTANCE:**

EHEC harbors a T6SS, which in other bacteria play a role in intestinal colonization and pathogenesis. T6SS is repressed *in vitro* in EHEC, making its study difficult, but it has been suggested to be required for full pathogenesis of EHEC. Here, we aimed to activate the T6SS *in vitro*. We found that activation of the bidirectional promoter is not enough for full T6SS expression, as a regulatory sequence downstream, named h3R, tightly regulates its expression. Deletion of the master regulator *hns* was not enough for full activation, and additional activation was required to produce some, but not all, proteins, causing a loss of bacterial fitness. These findings bring us closer to a full understanding of the mechanisms that control its expression. We predict novel regulation sites that will be necessary to fully understand the activation mechanisms and, thus, the function of T6SS in EHEC and perhaps other bacteria.

## INTRODUCTION

Among the different *Escherichia coli* pathotypes, EHEC serotype O157:H7 is particularly important because it encodes a Shiga-like toxin (Stx1 and Stx2), an AB_5_ toxin that binds to Gb3 receptors to enter the cell, where it halts protein translation by deadenylating 28S rRNA at position 4,324 (1, 2). Stx also activates pro-apoptotic pathways and the inflammasome (3), causing hemorrhagic enterocolitis. When the Stx reaches the blood, it disrupts organs with high expression of Gb3, such as brain, heart, and kidneys, leading to hemolytic-uremic syndrome (HUS). Approximately 15% of EHEC-associated gastroenteritis cases lead to HUS (4), and the annual estimated number of EHEC infections worldwide is 2.8 million (5), reflecting the importance of understanding the mechanisms of pathogenesis to design prevention alternatives. In addition to Stx, EHEC possesses diverse secretion systems that secrete and/or translocate effectors. Type 1 and Type 2 secretion systems (T1SS and T2SS) secrete hemolysin A (HlyA) and metalloprotease StcE, respectively (6–8), whereas autotransporter EspP and other adhesins are secreted by a T5SS (9). The T3SS injectisome is used to translocate proteins into the host cell cytoplasm, and it is important in bacterial adherence and in the manipulation of the host cell processes (10). Finally, EHEC encodes a T6SS, a nanomachine that resembles a poisoned spear and is activated by a spring-like mechanism to stab and kill neighboring cells (11).

The T6SS is composed of a spear-like structure formed by a hollow pole (Hcp) and a spearhead (VgrG), which is enveloped by a contractile sheath formed by TssB and TssC. The spear and sheath are anchored to the membrane by a baseplate (12, 13), and a membrane complex serves as a channel between the cytoplasm and the outer membrane (14). Diverse T6SS substrates are loaded by binding to Hcp or VgrG (15) and are translocated into the recipient cell upon sheath contraction, which can be other bacteria or an eukaryotic cell, while the sheath is recycled by the ATPase ClpV to fire again. To interact with the VgrG tip of the T6SS, proteins with a PAAR motif act as adaptors that are essential for T6SS function (16). Some PAAR proteins are long proteins with Rhs repeats (for Recombination hotspot) that form a shell-like structure and have a C-terminal domain, which brings function variability to the effectors (17). In bacterial competition, T6SS substrates include diverse bacterial toxins, such as mureinases, phospholipases, and nucleases, among many others (18), which impair bacterial homeostasis and cause cell death, helping attackers to invade an already occupied niche, such as the gastrointestinal lumen (13, 19). In addition to its antibacterial activity, T6SS has also been implicated in pathogenesis as it can translocate effectors directly into the host cells. These effectors include proteins with an actin cross-linking domain in *Vibrio cholerae* (20), proteins with a ROS-toxic domain in *Klebsiella pneumoniae* (21), and a trans-kingdom RNase named TseR from *Yersinia pseudotuberculosis*, which affects both prokaryotic and eukaryotic cells (22), among others (23–25). In *E. coli*, three subtypes of T6SS have been described (T6SS-1 to T6SS-3), and the most prevalent is the T6SS-2, which is present in EHEC and other STEC (26). Interestingly, the only reported EHEC effector is a catalase KatN that improves cell viability inside macrophages, although *katN* deletion was not sufficient to reduce mortality in a murine model, while T6SS mutation increased mice survival (27), suggesting that other T6SS effectors could be involved in EHEC pathogenesis, which is also supported by a strong association of *clpV* in STEC isolated from humans (28). Our previous data propose the Rhs-PAAR proteins encoded in the EHEC genome that could act as potential T6SS substrates. In EHEC EDL933, there are seven different Rhs-PAAR proteins encoded inside the T6SS island, as part of *vgrG* island, or as orphan genes, and these proteins harbor different C-terminal regions, including one with a RNase motif and some with structural homology to other bacterial toxins (29).

Multiple studies have established the complex inter-relation between EHEC and the microbiota (30–32), and several factors that impact niche occupancy are required for the establishment of EHEC infection (33), suggesting that the T6SS could play an important role in disease establishment. In laboratory conditions, the T6SS of EHEC is repressed by the H-NS protein (34), a histone-like protein that remodels the chromatin in the nucleoid and is responsible for the negative regulation of many virulence genes in pathogenic bacteria (35–37). Deletion of *hns* in EHEC results in higher levels of T6SS mRNAs (34), but *hns* deletion has also been shown to severely impact bacterial fitness and lead to changes in pathogenicity (38, 39), highlighting the need for a better model to study the T6SS *in vitro* in EHEC and other *E. coli*. The divergent promoter between *z0264* (*tssB*) and *z0265* has several transcription start sites (40), and at least one Rhs binds to mRNA to regulate the transcription of genes involved in pathogenesis (41). Nonetheless, more work is needed to fully understand the mechanisms that alleviate H-NS repression and allow T6SS expression and activation, which would allow a complete understanding of the role of the T6SS in pathogenic bacteria, whether for bacterial competition or trans-kingdom effector translocation. In this work, we aimed to induce T6SS by swapping the bidirectional promoter of the intergenic region between *tssB* and *hcp3*. Upon transformation, we detected higher levels of T6SS mRNA and determined that the *tssB* region is a single polycistronic operon, while the Hcp3 region contained at least two separate operons. Bacteria with swapped promoters did not secrete T6SS substrates, had no antibacterial activity, and did not translocate Hcp3 into the cytoplasm of HEp-2 cells, suggesting incomplete activation of the T6SS. By bioinformatic analysis, we found a region downstream of *hcp3* that contained a transcriptional terminator and a promoter region, which we renamed here as h3R. By deleting regions of the h3R, we observed a merging of the *hcp3-vgrG3* genes into a single operon, as well as a protector activity in the mRNA of *hcp3*, perhaps due to the formation of a hairpin in the *hcp3* 3′ UTR. We also show that mutation of *hns* is worse for expressing T6SS proteins than promoter swapping, and bacterial growth was impaired. EHECΔ*hns* activation on DMEM or modified M9 increased some, but not all, T6SS proteins and completely changed the secretion of hallmark proteins of EHEC. Finally, we suggest that additional transcriptional activation in the 3′ region of *paar* further complicates the regulation of the T6SS in EHEC. We pave the way for achieving the full expression of T6SS, which will allow the study of the function of the different effectors, thus providing new therapeutic targets to treat STEC infection, as well as other pathogenic *E. coli*.

## MATERIALS AND METHODS

### Bacterial strains

The strains and plasmids used in this study are listed in Table S1. Strains were grown in lysogeny broth (LB) (42), and supplemented with ampicillin (100 µg/mL), kanamycin (50 µg/mL), or chloramphenicol (10 µg/mL) when necessary. The plasmid pFCcGi (43) was a gift from Sophie Helaine and David Holden (Addgene plasmid #59324; http://n2t.net/addgene:59324; RRID:Addgene_59324). The plasmid pRL128 was a gift from Eric Cascales (Addgene plasmid #40180; http://n2t.net/addgene:40180; RRID:Addgene_40180). The plasmid *ilux* pGEX(-) (44) was a gift from Stefan Hell (Addgene plasmid #107879; http://n2t.net/addgene:107879; RRID:Addgene_107879). The plasmid pRetroX-Tight-MCS_PGK-GpNLuc (45) was a gift from Antonio Amelio (Addgene plasmid #70185; http://n2t.net/addgene:70185; RRID:Addgene_70185).

### Isogenic mutant construction

Genes *hns*, *tssM*, and *clpV* were replaced by a kanamycin-resistance cassette or a chloramphenicol-resistance cassette (*kanR* or *cmR*) using homologous recombination (46). Briefly, the *kanR* cassette was amplified by PCR using primers with flanking regions homologous to those of the genes to be replaced (Table S2). PCR products were purified and electroporated into EHEC + pKD46 previously induced with 1% arabinose. Kanamycin-resistant bacteria were screened by PCR using primers to corroborate *kanR* insertion and the deletion of the gene. pCP20 was used to remove *kanR* when necessary.

### Promoter swapping

The WT promoter of the T6SS (t6sP) from EHEC was swapped to a TacP-AraBAD double promoter, as previously described (47). Briefly, electrocompetent bacteria were transformed with a PCR product containing homologous regions to the 5′ UTR of *tssB* and *hcp3*, along with a *kanR* flanked by an AraBAD promoter and a Tac promoter. Colonies of kanamycin-resistant bacteria were screened by PCR to confirm the promoter swapping, and pCP20 was used to remove the *kanR* cassette.

### Chromosomal deletion of region I of h3R

The *hcp3*-h3RΔI-*vgrG3* region was amplified from pTrc:Hcp3-h3RΔI-VgrG3 using primers hcp3-RT-F and vgrG3-RT-R, and then purified. Region with the swapped promoters and the *kanR* cassette was amplified from EHEC::t6sP::LA using primers tssB-RT-R and hcp3-RT-R, then purified. Both amplicons were fused by overlapping PCR, and the full fragment (*tssB*-TacP-*kanR*-AraBAD-*hcp3*-h3RΔI-*vgrG3*) was used for promoter swapping. Deletion of region I was confirmed by PCR.

### RNA extraction and qPCR

Overnight (ON) cultures of bacteria were diluted 1:50 and grown until exponential phase (0.5–0.6), then centrifuged at 3,000 × *g*. RNA was obtained with TRIzol (ThermoFisher) according to the manufacturer’s instructions. Manufacturer’s instructions were followed, and the final pellet was resuspended in 50 µL of diethyl decarbonate-treated water. RNA was incubated with DNase I (Roche) at 25°C for 2 h, then converted to cDNA using M-MuLV RT (NEB). Assays of qPCR were performed using Taq Polymerase (ThermoFisher) with EvaGreen (GoldBio) according to the manufacturer’s instructions. RNA values were analyzed with Bio-Rad CF96X Manager, corrected with 16S RNA, then normalized with WT EHEC.

### Gene cloning

Primers for cloning are listed in Table S2. The genes *hcp3*, *vgrG3*, *paar*, *clpV*, and *tssB* and the region between *hcp3 and vgrG3* were cloned from EHEC EDL933. The *gfp* and *mCherry* genes were cloned from pFCcGi. The *nluc* gene was cloned from pGFP-Nluc. Plasmid pTrcHis2B was used as backbone, and a 4Cys flag was added to create pTrc-4C6H, which served as the backbone for most constructions. AmpR-oriC region was interchanged for p15A-cmR using overlapping PCR to create p15A-Trc.

### Protein expression and antibody generation

Hcp3, VgrG3, PAAR, ClpV, and TssB were purified using His-tag affinity chromatography under denaturing conditions with Ni-NTA Agarose (Qiagen, ID 30,210), following the manufacturer’s instructions. Samples were further purified using electroelution and quantified. Mice were injected intramuscularly with 1.5 µg of protein along with TiterMax adjuvant. Mice were immunized three times over a 42-day period. After the third immunization, blood was harvested, and the serum was directly used in Western blot assay.

### Western blot

Protein was obtained from total bacterial lysates, and 50 µg was separated by SDS-PAGE. Proteins were transferred to PVDF membranes using Trans-Blot SD Semi-Dry Transfer Cell. Membranes were blocked with PBS containing 0.05% Tween (PBST) and 5% non-fat dry milk for 1 h with vertical agitation at 22 rpm. Primary antibodies were incubated ON at 4°C. Membranes were washed three times with PBST, then incubated with secondary antibodies bound to HRP (Invitrogen, Cat. G21040), or a mix of secondary antibodies bound to biotin along with Streptavidin-AlexaFluor 680 or 780 (Jackson ImmunoResearch, Cat. 115-065-008, Cat. 111-065-003, Cat. 016-650-084, and Cat. 016-620-084) at a 1:20,000 dilution in PBST for 1 h. Membranes were washed three times, then visualized on an Odyssey FC imager, using Immobilon Western HRP Substrate (Millipore, Cat. WBKLS0500) when necessary. Anti-DnaK was used as a loading control (Invitrogen, Cat. #PA5-117658). Anti-Calnexin and anti-GAPDH were acquired from ABclonal (Cat. A4846 and AC002).

### Secretion assay

Overnight cultures of bacteria were diluted 1:25 and grown until exponential phase (0.5–0.6), then centrifuged at 3,000 × *g*. Supernatants were filtered using 0.22 µm membranes to remove the remaining bacteria. Supernatants were then precipitated using 15% trichloroacetic acid (TCA) ON at 0°C. Supernatants were centrifuged at 8,000 × *g* for 1 h, then washed with ice-cold acetone, and centrifuged again for 30 min. Proteins were resuspended in 1 M Tris-HCl, pH 8.8, and Laemmli buffer was added to a final concentration of 1×. Samples of 3 mL of supernatant were concentrated to 50 µL and separated in SDS-PAGE. Bovine serum albumin (BSA) was used as precipitation control.

### Secretion assay using LUMIO or Nluc

For LUMIO assay, 4×Cys flag was fused at the C-terminal of Hcp3 and expressed in a pTrc plasmid, while for Nluc assay, Nluc was fused at the C-terminal of VgrG3 or PAAR and expressed in a pTrc plasmid. Overnight cultures were diluted 1:50 and grown until exponential phase, then centrifuged, and the supernatant was filtered as mentioned above. LUMIO (ThermoFisher) was added for Hcp3, and fluorescence at 480/508 nm was measured for 1 h at 37°C. Furimazine (Promega) was added for VgrG3-Nluc and PAAR-Nluc, and luminescence was immediately quantified.

### Bacterial competition

ON cultures of bacteria were diluted 1:25 in fresh media and incubated for 3 h. Optical density at 600 nm (OD_600_) was measured and adjusted to 0.5. A 4:1 mix (predator:prey) was spotted thrice in LB media supplemented with 0.1 mM IPTG and 0.2% arabinose, then incubated ON at 30°C. Agar spots were resuspended in PBS and serially diluted in LB with the appropriate antibiotic to recover the prey and calculate the colony-forming units (CFUs).

### Cell culture and infection model

HEp-2 (ATCC CCL-23) cells were cultured using DMEM supplemented with 10% fetal bovine serum (HyClone, Logan, UT), 1% non-essential amino acids, 5 mM glutamate, penicillin (100 U/mL), and streptomycin (100 µg/mL). At an 80% confluency, cells were infected with WT EHEC, t6sP::LA, or t6sP::LAΔ*tssM* at a multiplicity of infection (MOI) of 10. Infection was carried out for 3 h at 37°C and 5% CO_2_.

### Cell fractionation

After the infection, HEp-2 cells were washed three times with PBS, then physically detached and resuspended in FT buffer (20 mM Tris-HCl, KCl 600 mM, 20% glycerol, and cOmplete Protease Inhibitor 1×). Cells were freeze-thawed three times, then centrifuged 15 min at 13,000 × *g*. The supernatant (cytoplasmic fraction) was recovered, and the pellet was washed three times with PBS, then resuspended with RIPA buffer (50 mM Tris HCl, 150 mM NaCl, 1% Nonidet P-40, 0.5% Sodium deoxycholate, cOmplete Protease Inhibitor 1×). The pellet was resuspended by vertical agitation for 30 min at 4°C. The solution was centrifuged for 15 min at 13,000 × *g*, and the supernatant (Membrane fraction) was recovered. Samples were quantified, 1× Laemmli buffer was added, and samples were incubated 10 min at boiling temperature. Samples were stored at −20°C.

### Bioinformatic analysis

Transcriptional termination prediction was performed using iTerm-PseKNC (48). Transcription factor binding site prediction and promoter prediction were performed using Virtual Footprint (49) and Sapphire (50). RNA secondary structure prediction was performed using RNAfold (51).

### Reporter analysis of the t6sP using GFP and mCherry

The bidirectional T6SS promoter was cloned between GFP and mCherry, which functioned as reporters for *tssB* and *hcp3,* respectively. Fluorescence of ON cultures was normalized to WT EHEC and to OD_600_.

### Reporter analysis of the h3R region

The region between *hcp3* and *vgrG3* genes (h3R) was cloned in pTrc-4C6H downstream the *gfp* gene and directly upstream of *mCherry*. EHEC bacteria harboring pGFP-h3R-mCherry was grown for 6 h at 37°C in different media, and measurements of GFP fluorescence, mCherry fluorescence, and OD_600_ were taken every 30 min.

### Reporter analysis using *ilux*

Bidirectional promoters for *hcp3* or *tssB* were cloned in pGEX:*ilux*. EHEC with pGEX:hcp3P:*ilux* or pGEX:tssBP:*ilux* was incubated in different media, and bioluminescence was measured for 6 h in a white opaque microplate. For h3R, *ilux* was cloned downstream of the *hcp3*-h3R-*vgrG3* region.

### Expression of T6SS in EHECΔ*hns* in DMEM and MM9

Overnight cultures were diluted 1:20 in fresh MM9 and grown 4 h at 37°C and 5% CO_2_ (activation). Then, OD_600_ was measured, and bacteria were resuspended in fresh MM9 or DMEM culture at an OD_600_ of 0.05. Bacteria grew for 3 h, after which supernatants were obtained by centrifugation, filtered with 0.22 µm membranes, and precipitated with TCA.

## RESULTS

### Swapping the unknown bidirectional promoter increases T6SS mRNA but not full protein expression

**Fig 1:**
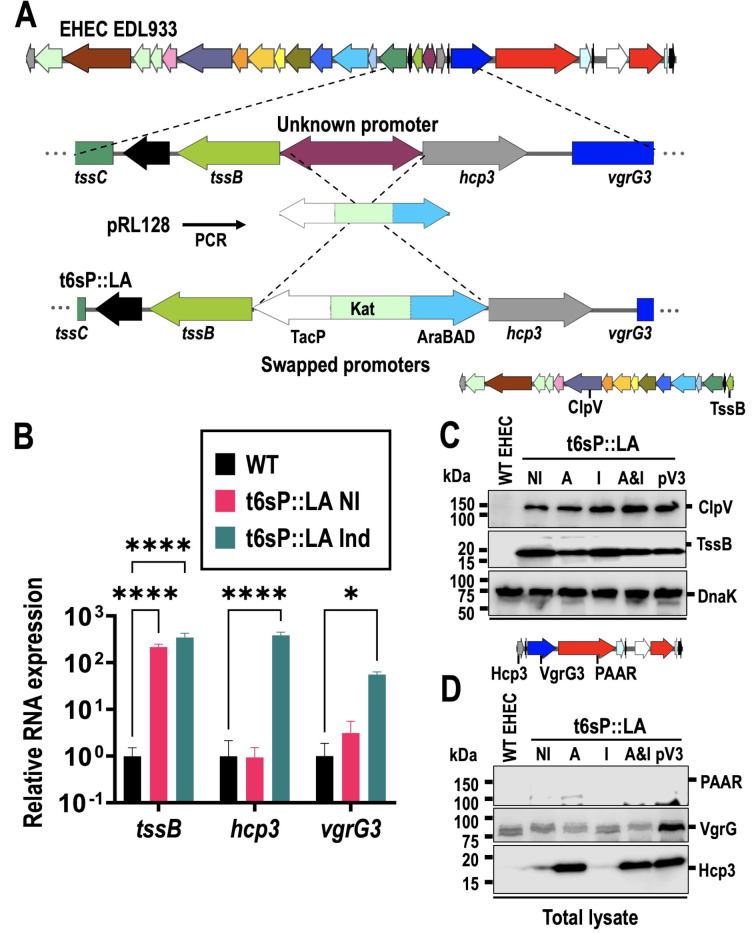
Bidirectional promoter swapping for EHEC EDL933 increased mRNA expression of T6SS-related genes, but not all proteins were detected. (**A**) Map of the promoter region of the T6SS and promoter swapping. (**B**) Relative RNA expression in WT EHEC vs EHEC::t6sP::Lac-Ara (t6sP::LA) not induced (NI) or induced (Ind: IPTG/Ara). (**C**) Expression of T6SS proteins in t6sP::LA from genes in the *tssB* operon (NI, not induced; A, arabinose 0.2%; I, 0.2 mM IPTG; A & I, 0.2% arabinose and 0.2 mM IPTG; pV3, bacteria with plasmid expressing VgrG3 as well as induction with 0.2% arabinose and 0.2 mM IPTG). (**D**) Expression of T6SS proteins in t6sP::LA from genes in the *hcp3* operon. Bacteria were grown until OD of 0.6 in LB with 0.2 mM IPTG and 0.2% arabinose, then RNA was obtained with TRIzol, treated with DNase, and converted into cDNA. RNA expression was normalized with 16S. The plots were made using at least three independent replicates. Statistical analysis was performed using one-way ANOVA followed by Dunnett’s multiple comparisons test. (*, *P* < 0.05; ****, *P* < 0.0001). Total lysates (50 µg) were separated in an SDS-PAGE, then transferred to PVDF, and Western blot was performed to detect Hcp3, TssB, ClpV, VgrG3, and PAAR. For panel A, different colors were used to represent the structural and effector proteins, and related proteins were displayed with the same color (blue for *vgrG*; light green for *tssA*-like genes; gray for *hcp*; red for *rhs*; light turquoise for the putative immunity genes downstream of an *rhs* gene).

The T6SS in EHEC is turned off in laboratory conditions, and other studies in EHEC have determined that *hns* is bound to the region between *hcp3* and *tssB* (27, 40). While deletion of *hns* can overcome the repression, H-NS also plays an important role in pathogenesis and bacterial fitness (52–54). The T6SS island comprises two operons: one starting with *tssB* and comprising structural genes such as *tssC*, *tssM,* and *clpV*, while the other operon starts with *hcp3* and includes potential T6SS substrates, such as *vgrG3* and *paar*, as well as its cognate immune protein encoded in *paarI* (29). As an alternative to *hns* mutation, we investigated swapping the unknown bidirectional promoter between *hcp3* and *tssB* for two known promoters (AraBAD and TacP, responsive to arabinose and IPTG, respectively) (Fig. 1A). The resultant bacteria were EHEC::t6sP::Lac-Ara (from here on called t6sP::LA), which contained higher levels of transcripts from *tssB*, *hcp3*, and *vgrG3* upon induction, although *tssB* levels were already higher in uninduced conditions compared to WT EHEC, due to the leaky tac promoter (Fig. 1B). Interestingly, we observed that *vgrG3* expression was not as high as *hcp3* expression under induced conditions, suggesting a regulatory region between *hcp3* and *vgrG3* that weakens RNA transcription downstream of *hcp3*.

To corroborate RNA transcription and analyze substrate secretion, we generated a series of polyclonal antibodies against proteins encoded in the *tssB* operon (TssB and ClpV), as well as proteins encoded in the other direction of the island, the *hcp3* operon (Hcp3, VgrG3, and PAAR). Expression of T6SS proteins in WT EHEC lysates could not be detected, as the T6SS island is supposed to be directly inhibited by H-NS. In contrast, TssB and ClpV were detected in cell lysates of t6sP::LA under both non-induced and induced conditions, although expression was higher with IPTG induction (Fig. 1C). The *hcp3* operon was swapped for an AraBAD promoter, which has tighter regulation than Tac, and Hcp3 expression was confirmed in bacterial lysates upon arabinose induction, but not in the absence of arabinose or with IPTG (Fig. 1D). However, neither VgrG3 nor PAAR proteins were detected even upon induction with arabinose and IPTG, which suggests a mechanism for gene inhibition downstream of *hcp3*. Since VgrG3 was clearly detected in bacterial lysates of t6sp::LA carrying a plasmid with the *vgrG3* gene under a TrcP promoter, Hcp secretion to the supernatant—a hallmark of T6SS activation—was examined. We looked for Hcp3 in the supernatant, as well as other possible substrates such as VgrG3 and Rhs-PAAR proteins. Secretion of T6SS substrates was not detected in the supernatant, while Tir, a T3SS substrate, was clearly detected (Fig. S1A), suggesting that something is obstructing T6SS function. Furthermore, while VgrG3 expression in t6sp::LA with a plasmid containing *vgrG3* gene was confirmed in bacterial lysates, no secretion of VgrG3 was found, and its expression did not complement the phenotype for secretion of PAAR, VgrG3, or Hcp3. Since the protein expression could be low, but enough for T6SS function, the Hcp3 detection was enhanced by monitoring the secretion of Hcp3 with a 4-Cys tag that produces fluorescence in the presence of LUMIO. Again, no difference was found in t6sP::LA and its isogenic mutant in *tssM*, an essential gene for T6SS function (Fig. S1B). A fusion of the *vgrG3* or *paar* genes with Nluc was also used to measure protein secretion, but no difference was found between t6sP::LA and its isogenic mutant in *clpV* gene, another essential gene for T6SS function (Fig. S1C). VgrG3 expression was not observed in bacterial lysates, even when promoter swapping was performed so that *hcp3* was under control of the stronger Lac promoter (Fig. S1D). Together, these results suggest that even if some of the proteins of the T6SS are being expressed, VgrG3 and PAAR cannot be detected in bacterial lysates, and no secretion of T6SS substrates was occurring.

### Promoter swapping did not display antibacterial activity or effector translocation in host cells

**Fig 2:**
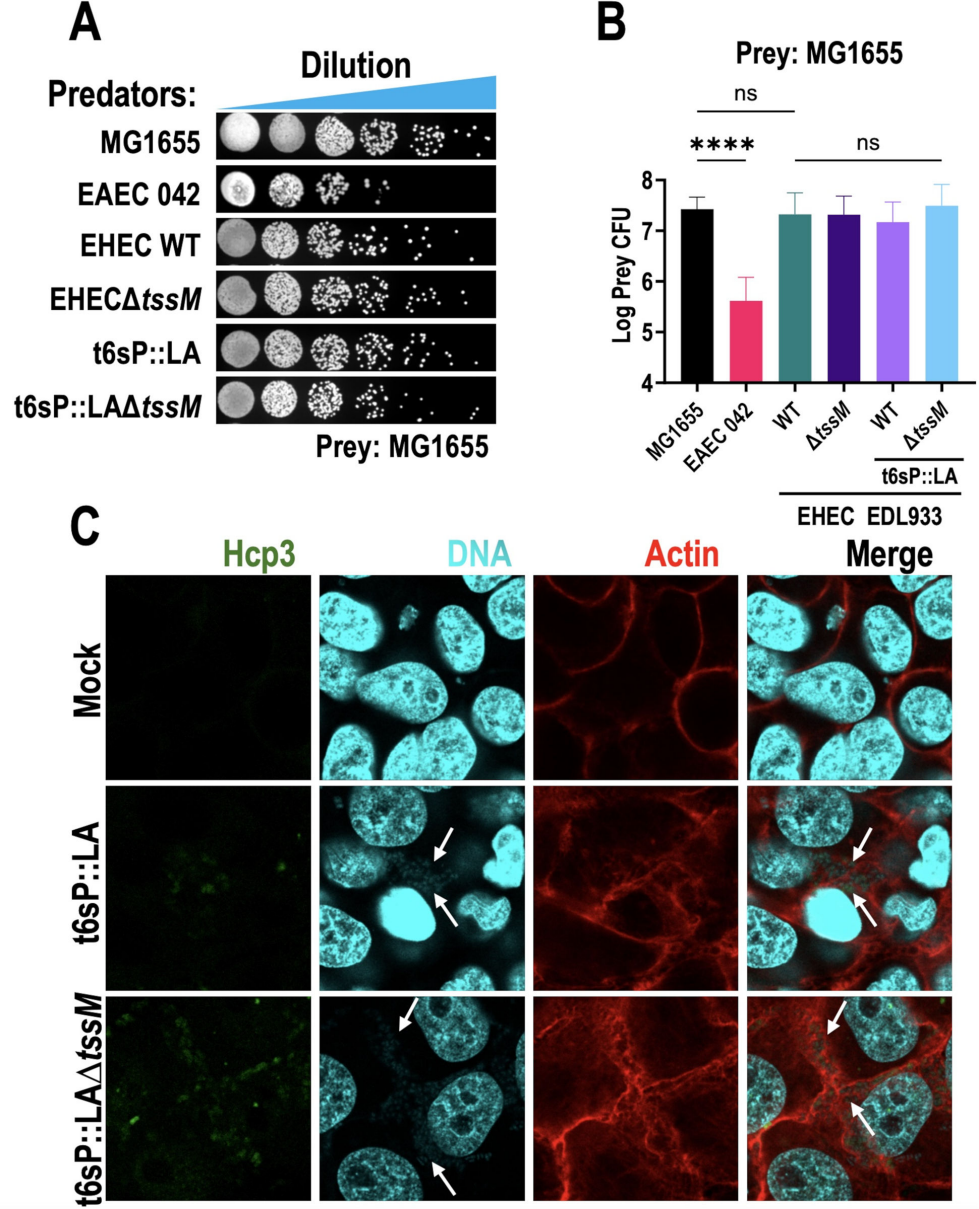
No antibacterial activity nor effector translocation of Hcp3 was observed after promoter swapping. (**A**) Prey dilutions in presence of different predators. (**B**) Prey CFUs recovered in the presence of different predators. (**C**) Immunofluorescence of HEp-2 cells infected with WT EHEC, t6sP::LA, or t6sP::LAΔ*tssM*. For bacterial competition, bacteria were grown in LB at 37°C until an OD of 0.8–0.9, then adjusted to 0.5 and mixed in a 4:1 ratio (predator:prey). Samples of 20 µL were spotted into LB plates supplemented with 0.2% arabinose and 0.1 mM IPTG and incubated ON at 30°C. Spots were then resuspended in PBS and serially diluted to obtain countable colonies. The plots were made using the means of at least three independent experiments. A Kruskal-Wallis test was performed followed by a Dunn’s correction to estimate statistical significance. (ns, not significant; ****, *P* < 0.0001). For infection assays, EHEC and its variants were induced with 0.2 mM IPTG and 0.2% arabinose in MM9 for 4 h, then bacterial cells were washed and resuspended in DMEM. HEp-2 cells at 80% confluence were fasted ON and then infected with a MOI of 10 for 4 h. For immunofluorescence, cells were fixed with 4% PFA and incubated with Anti-Hcp3. Anti-IgG-Mouse coupled with biotin was used as a secondary antibody, and 5-(4,6-dichlorotriazinyl)aminofluorescein was used to bind to biotin. F-actin was stained using Rhodamine Phalloidin, and DNA was stained using Hoechst.

Although substrate secretion could not be detected, the T6SS still could be activated in the presence of other bacteria for antibacterial activity. To test the antibacterial activity of T6SS in EHEC or t6sP::LA, bacterial competition assays were conducted. Enteroaggregative *E. coli* (EAEC) str. 042 was used as a positive control, as this strain possesses the three subtypes of T6SS found in *E. coli*, and another strain (EAEC 17-2) has shown antibacterial activity linked to T6SS-1 and T6SS-3 (55). EAEC 042 displayed antibacterial activity, reducing prey recovery by two logarithms, while neither WT EHEC nor t6sP::LA displayed reduced prey survival, indicating no antibacterial activity after promoter swapping (Fig. 2A and B). Prey recovery was similar in WT EHEC and EHECΔ*tssM*, indicating that under these conditions EHEC has no T6SS-dependent antibacterial activity. Moreover, trans-expression of both VgrG3 and PAAR-PAARI did not result in antibacterial activity (Fig. S2A). Promoter swapping was attempted with other bacteria, such as *E. coli* str. HS or EHEC O157:H7 str. Rafaela II, which possesses a t6sP very similar to EHEC str. EDL933 (Fig. S2B), but no antibacterial activity was observed with the WT bacteria or after promoter swapping. Trans-expression of both VgrG3 and PAAR-PAARI also did not result in antibacterial activity. EASTEC O104:H4 also possesses a T6SS-2 very similar to EHEC str. EDL933, and no antibacterial activity was found in WT EASTEC O104:H4, although no experiments with swapped promoters were conducted.

As some of the T6SS only translocate effectors into eukaryotic cells, we next searched for translocation of Hcp3 into HEp-2 cells during infection. The bacteria t6sP::LA and t6sP::LAΔ*tssM* adhered to HEp-2 cells and produced the characteristic pedestals seen in EHEC, confirming translocation of effectors of the T3SS (Fig. 2C). Hcp3 signal was observed inside the bacteria, but no signal was observed inside the HEp-2 cells. We also looked for Hcp3 or VgrG3 translocation by Western blot in the cytoplasmic and membrane fractions of HEp-2 cells infected with t6sP::LA, but no signal was observed (Fig. S2C). Together, these findings suggest that t6sP::LA can cause A/E lesions in HEp-2 cells using the T3SS, which is a hallmark of EHEC pathogenesis, but it does not translocate Hcp3 into the HEp-2 cells using the T6SS and therefore no T6SS-associated damage was observed, even though expression is detectable inside the bacteria.

### Structural genes are encoded in a polycistronic operon, but effector genes are in different operons

**Fig 3:**
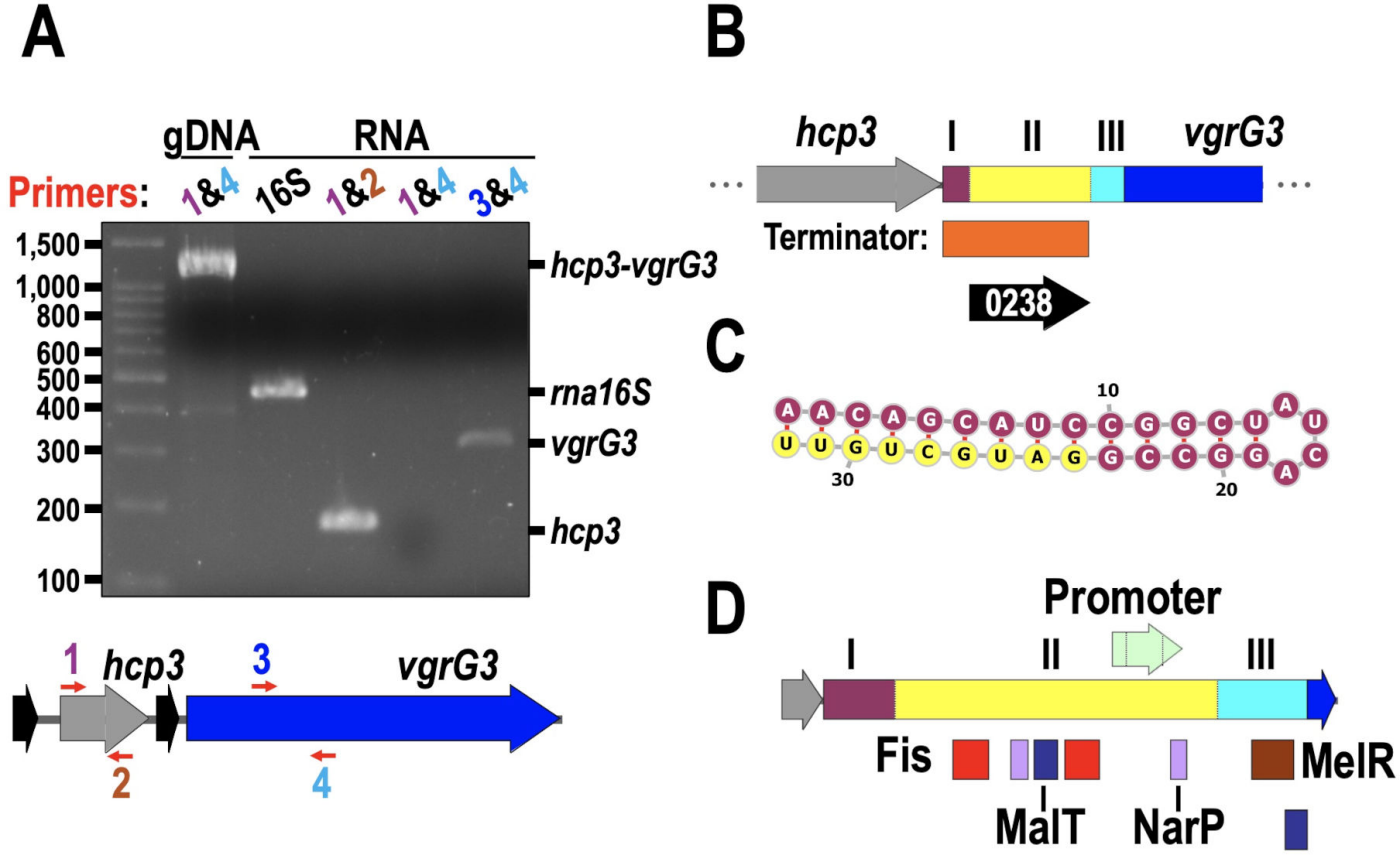
Messenger RNA showed that *hcp3* and *vgrG3* are encoded as different operons. (**A**) End-point RT-PCR using different combinations of primers for *hcp3* (1 and 2) and *vgrG3* (3 and 4) genes using overlapping primers. (**B**) Terminator sequence found 3′ UTR of *hcp3*. (**C**) Hairpin prediction on the *hcp3* 3′ UTR region. (**D**) Promoter prediction and transcription factor binding site prediction in the *hcp3* 3′ UTR region. RNA was amplified using specific primers (red arrows), and gDNA was used as a control. The transcriptional terminator was predicted using iTerm-PseKNC. The secondary structure of the *hcp3* 3′ UTR was modeled and visualized with RNAfold. Regions I and II of the h3R were colored as yellow and plum in panels B to D, while region III was colored turquoise. Promoter prediction was performed using BPROM, Sapphire, and Virtual Footprint. Only scores with 90% of the max score were considered. Boxes are colored for the transcription factor predicted (red for Fis, lavender for NarP, indigo for MalT, and brown for MelR).

As protein translation was not observed for genes downstream of *hcp3*, we analyzed the region between *hcp3* and *vgrG3*. Firstly, we wondered if *hcp3* and *vgrG3* were encoded as different operons, so we amplified RNA using both internal primers for *hcp3* and *vgrG3*, as well as primers spanning from *hcp3* to *vgrG3*. While individual *hcp3* and *vgrG3* amplicons were detected, no product was obtained for both *hcp3-vgrG3* genes from mRNA, although amplification was successful from genomic DNA. This indicates that *hcp3* and *vgrG3* are encoded as separated operons (Fig. 3A), unlike the *tssB* operon in which ClpV expression was detected and is located ten ORFs away from *tssB* (see Fig. 1C).

### An intergenic region between *hcp3* and *vgrG*3 acts as a regulatory sequence (h3R)

By analyzing the *hcp3* 3′ UTR with iTerm-PseKNC software, we found a transcriptional terminator directly after the *hcp3* STOP codon (Fig. 3B), suggesting that RNA expression is terminated after *hcp3*, even if the *hcp3* promoter is swapped. A secondary structure of RNA was predicted at position +8 of the *hcp3* 3′ UTR, forming a 32-base loop, which could act as a transcription terminator (Fig. 3C). The presence of a terminator also suggests that a different promoter must act as a binding site for the RNA-pol to start RNA synthesis to produce *vgrG3*. Indeed, a promoter sequence was found overlapping the 3′ region of EDL933_0238. A Virtual Footprint analysis revealed binding sites for Fis (two sites), MalT (two sites), NarP (two sites), and MelR (one site) (Fig. 3D). Collectively, these data strongly suggest that *vgrG3* is encoded as a separate operon from *hcp3* and has its own activation signal. As the *hcp3* 3′ UTR has its own terminator and promoter regions, we named h3R hereon and divided it into three fragments, where the region I has the terminator, the region II has the predicted promoter, and the region III comprises the *vgrG3* 5′ UTR.

**Fig 4:**
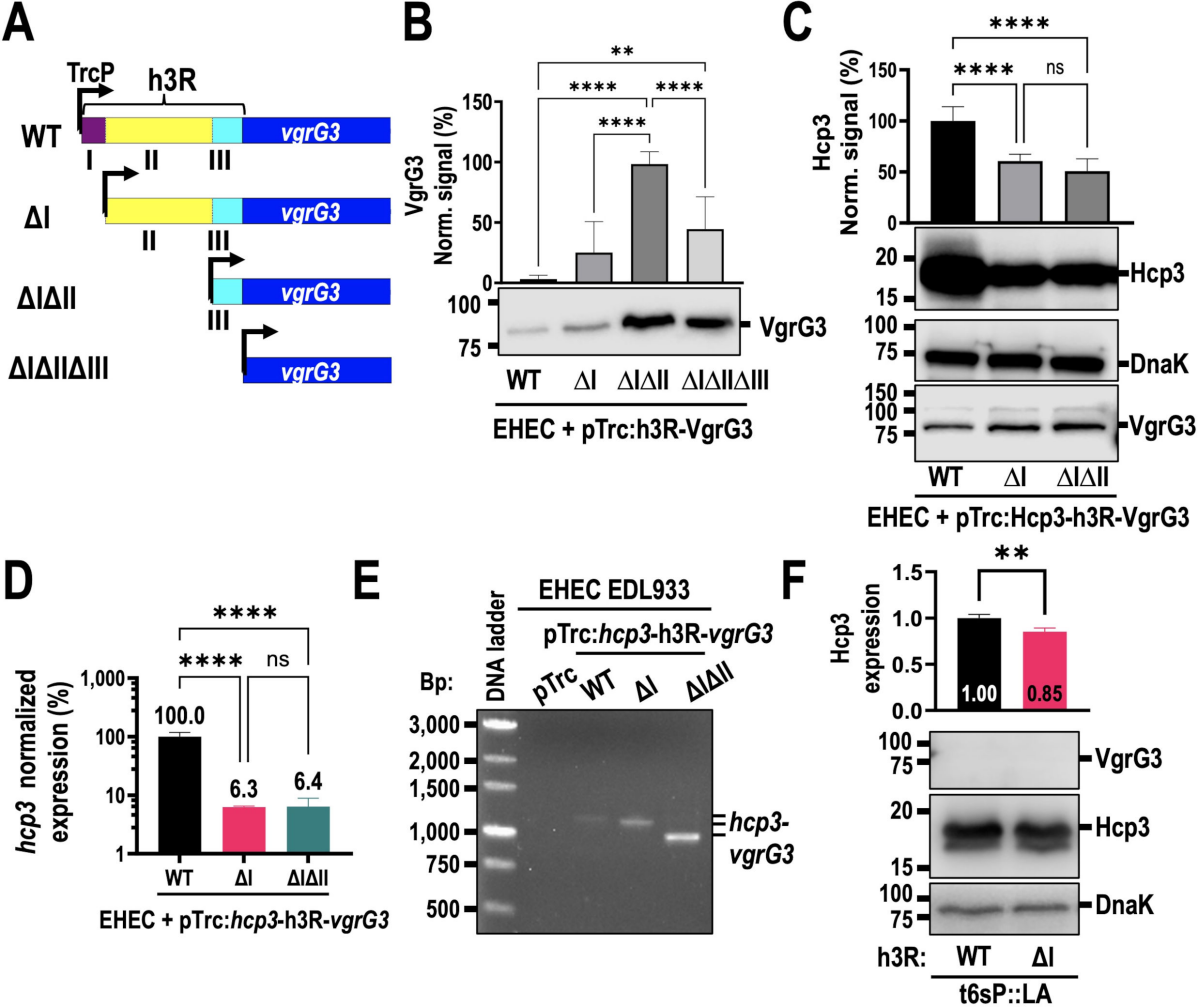
Regions I and II of the h3R actively regulate *vgrG3* and *hcp3* expression. (**A**) Deletion of regions I and II of h3R in a pTrc plasmid with *vgrG3*. (**B**) VgrG3 expression in EHEC using WT h3R, as well as mutants of regions I, I–II, or I–III (ΔI, ΔII, or ΔIΔIIΔIII). (**C**) Hcp3 and VgrG3 expression in plasmids with WT h3R vs mutants of regions I or I and II. (**D**) Normalized expression of *hcp3* mRNA in EHEC EDL933 + pTrc:Hcp3-h3R-VgrG3, using WT h3R, or its deletions in regions I or I and II. (**E**) Region containing *hcp3-vgrG3* was amplified from mRNA in EHEC with plasmids having WT h3R and mutants of regions I or I and II, along with *hcp3* and *vgrG3* under control of a Trc promoter. (**F**) Hcp3 and VgrG3 expression in t6sP::LA with a deletion of the region I of h3R on native chromosome locus. Bacteria were induced with 0.2 mM IPTG for 3 h, or with 0.2% arabinose and 0.2 mM IPTG for 4 h in t6sP::LA, then lysed and quantified. Western blot was performed with polyclonal antibodies to detect Hcp3 and 6xHis antibodies for VgrG3. For RNA analysis, RNA was obtained and converted to cDNA. *hcp3* expression was normalized against 16S rRNA and then against WT EHEC. Statistical analyses for panels B to D were performed using one-way ANOVA followed by Tukey’s multiple comparisons test (ns, not significant; **, *P* < 0.01; ****, *P* < 0.0001), and for panel F, Student’s *t*-test was performed (**, *P* < 0.01). All graphs represent the mean ± SD of at least three biological replicates.

As the h3R can interrupt the expression of a strong promoter such as TacP, we tried to establish if deletions in regions I or I and II could abrogate the inhibition, so we designed four plasmids with the WT h3R, and mutants of regions I or I and II, all preceded by a strong Trc promoter, while *vgrG3* was also cloned on the ATG of pTrc to represent a mutant for regions I, II, and III (Fig. 4A). Expression of VgrG3 was very low by Western blot when using WT h3R, but it was clearly detected in ΔIΔII h3R, indicating that the inhibitor for *vgrG3* extends to region II (Fig. 4B). Interestingly, VgrG3 expression was higher when region III was present, as opposed to ΔIΔIIΔIII. To corroborate these results, we then cloned *hcp3-*h3R-*vgrG3* and versions mutated in regions I or I and II of h3R for their comparison. VgrG3 expression was much lower when *hcp3* was present, and deletion of regions I or I and II brought only a small increase in VgrG3 detection. Interestingly, Hcp3 expression was 50% lower after deleting regions I or I and II (Fig. 4C). Furthermore, *hcp3* mRNA was also greatly reduced after deleting regions I or I and II, showing only 6% of mRNA compared to WT h3R (Fig. 4D). By amplifying mRNA from the *hcp3* to *vgrG3* region, we found that removing regions I and II caused higher expression of the intergenic mRNA compared to the WT, which was also observed by removing region I but in lower proportion. This mRNA was only slightly present in WT h3R from plasmid and not detectable in WT EHEC without plasmid (Fig. 4E). To discard any potential artifacts introduced by the plasmid expression, we performed the deletion of region I in t6sP::LA at the native chromosomal locus and observed that expression of Hcp3 was also reduced, albeit not as noticeable compared to plasmid expression (85% of expression, *P* = 0.0098 by Student’s *t*-test), whereas VgrG3 expression was below the detection limit (Fig. 4F). The lower detection of both mRNA and protein suggests that region I of h3R stabilizes the *hcp3* mRNA and prevents its degradation, although further experiments are required to discard other possibilities, such as secondary structures or polymerase arrest.

### The h3R regulatory sequence has contrary effects on *hcp3* and *vgrG3* expression

**Fig 5:**
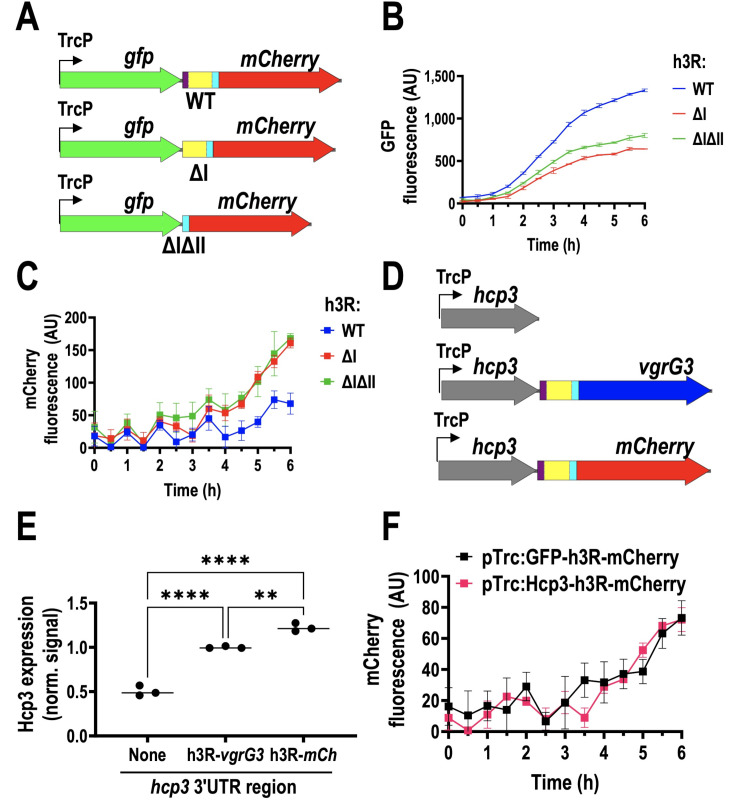
The h3R improves expression of upstream genes and inhibits downstream gene expression. (**A**) Schematic representation of three variants of gfp-h3R-mCherry with WT h3R as well as deletions of region I or I and II (WT, ΔI or ΔIΔII). (**B**) GFP expression in WT h3R region vs I or I and II mutants. (**C**) mCherry expression in WT h3R region vs I or I and II mutants. (**D**) Schematic representation of three plasmids with different 5′ UTR regions after *hcp3*. (**E**) Hcp3 expression in plasmids with different 3′ UTR regions after *hcp3*. (**F**) mCherry reporter for h3R region cloned after *gfp* or *hcp3*. h3R region was cloned after *gfp* or *hcp3*, and before *vgrG3* or mCherry, then regions I or I and II were deleted by PCR. For panels B, C, and F, overnight cultures were diluted 1:10 in fresh LB media with 0.2 mM IPTG and incubated 6 h at 37°C, measuring mCherry and/or GFP fluorescence every 30 min. Hcp3 expression was analyzed by Western blot. Total lysates (50 µg) were separated in an SDS-PAGE. The plots were made using at least three independent replicates. Statistical analysis for panel E was performed using one-way ANOVA followed by Tukey’s multiple comparisons test. (**, *P* < 0.01; ****, *P* < 0.0001).

Since the h3R regulatory sequence had contrary effects on *hcp3* and *vgrG3*, stabilizing one and inhibiting the other, we evaluated if this was a property specific to *hcp3* and *vgrG3* or intrinsic of h3R. Therefore, we cloned the h3R region between *gfp* and *mCherry* in a pTrc plasmid, including WT h3R as well as its deletions in regions I or I and II (Fig. 5A). Similar to Hcp3, GFP expression after 6 h was halved when the regions I or I and II of h3R were deleted, while the fluorescence of mCherry was doubled, confirming our previous results (Fig. 5B and C). To rule out possible interactions in the expression of *hcp3* by *vgrG3*, we tested Hcp3 expression in constructions where the 3′ UTR was removed entirely, against constructions with an intact h3R followed by *vgrG3* or *mCherry* (Fig. 5D). Hcp3 expression was halved when no h3R was present, while when h3R was present, Hcp3 expression was higher independently of the gene downstream of h3R, *vgrG3,* or *mCherry* (Fig. 5E). Similarly, whether *hcp3* or *gfp* was upstream of h3R, mCherry fluorescence was unchanged, indicating that regulatory regions are inside of h3R (Fig. 5F). When comparing the fluorescence of h3R-mCherry and its variants without an upstream gene, we found that deletion of regions I and II (ΔIΔII) produces higher levels of fluorescence than deletion of region I alone, but these two levels are still much lower than mCherry directly under the Trc promoter. This last result suggests that region III still inhibits mCherry expression, contrary to *vgrG3,* where the presence of region III resulted in higher expression (Fig. S3A and B, see Fig. 4B).

Since we had constructed a reporter system for h3R using mCherry, we also analyzed reporter expression under conditions that could activate h3R promoter region (see Fig. 3D) by using M9 media alternatively supplemented with glucose, IPTG, arabinose, melibiose, stachyose, mannose, lactose, galactose, xylose, arginine, alanine, aspartate, or asparagine. We found that only stachyose slightly augmented reporter fluorescence, but the increase was less than twofold, and poor growth was observed for EHEC (Fig. S3C).

### Promoter swapping is a better tool than *hns* deletion toward T6SS expression

**Fig 6:**
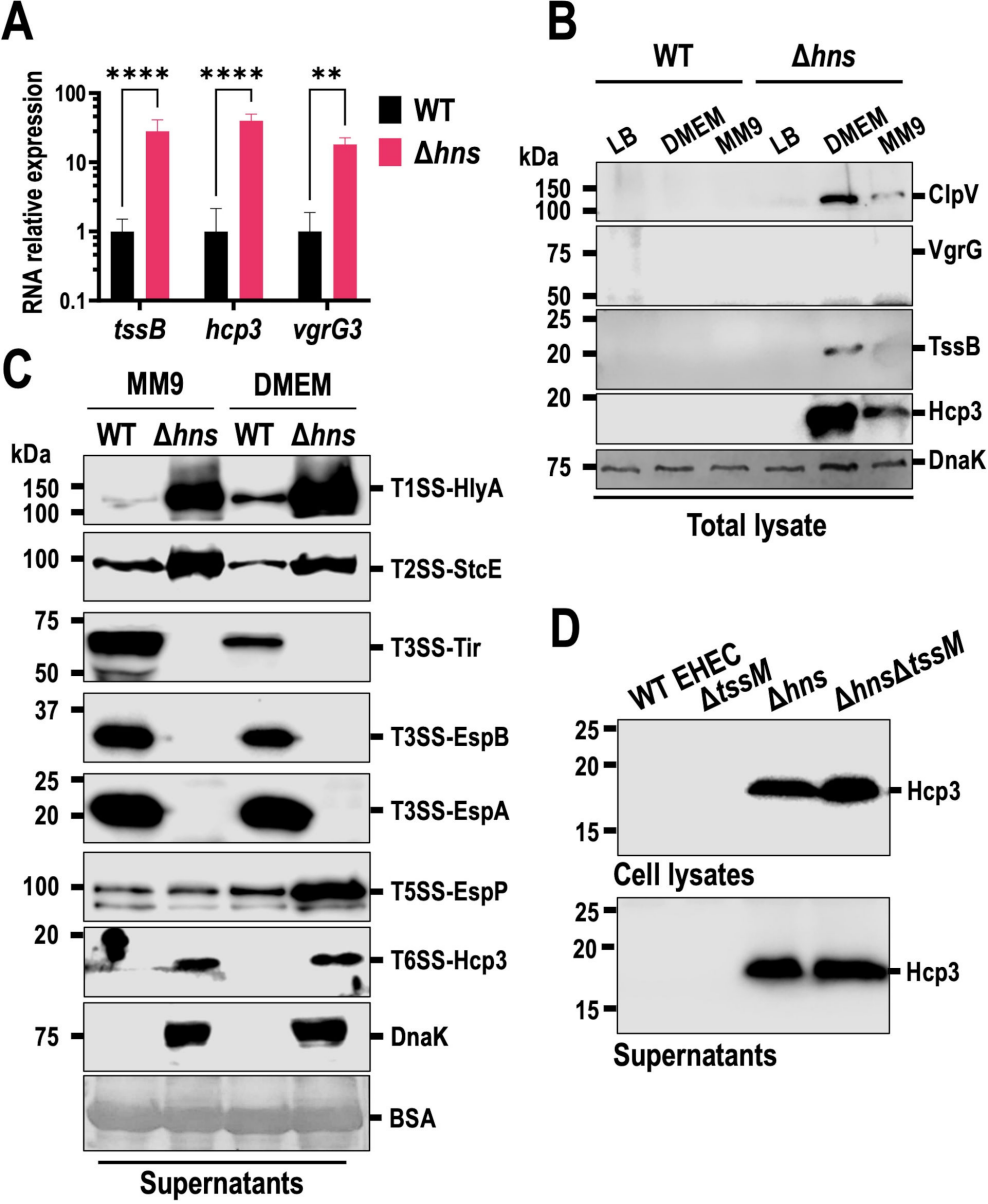
Deletion of the *hns* gene in EHEC increases transcription, but not translation of all T6SS genes, and dysregulates other secretion systems. (**A**) Relative mRNA expression of T6SS genes in WT EHEC and EHECΔ*hns* (Δ*hns*) in three biological replicates. (**B**) Expression of T6SS proteins in WT EHEC and EHECΔ*hns* grown in LB, MM9, and DMEM. (**C**) Protein secretion of different secretion systems in WT EHEC vs EHECΔ*hns*. (**D**) Expression and secretion of Hcp3 in WT EHEC, EHECΔ*tssM*, EHECΔ*hns,* and EHECΔ*hns*Δ*tssM* in MM9. For gene expression, RNA and proteins were extracted from EHEC and EHECΔ*hns* grown in LB at exponential phase (0.5–0.6). RNA was treated with DNase and converted to cDNA. RNA expression was normalized with 16S RNA, and data were obtained from at least three independent experiments. RNA expression was analyzed using two-way ANOVA followed by a Sidak multiple comparisons test. (**, *P* < 0.01; ****, *P* < 0.0001). For protein expression in LB, bacteria were grown for 4 h at 37°C in static conditions. Otherwise, bacteria were grown in MM9 for 4 h and then resuspended in fresh MM9 or DMEM media and incubated for 3 h at 37°C and 5% CO_2_. Proteins were extracted and separated by SDS-PAGE. Supernatants were filtered and then precipitated with TCA at a final concentration of 15%. Western blotting against proteins of diverse secretion systems was performed searching for HlyA for T1SS; StcE for T2SS; EspA, EspB, and Tir for T3SS; EspP as an example of autotransporter (T5SS), and Hcp3 as a T6SS substrate. DnaK was used as loading control and as a control for cytoplasm contamination. BSA was used as a loading control for precipitated supernatants.

As our attempts to activate the full-length T6SS in WT EHEC were unsuccessful, we tried to study T6SS expression and secretion in EHECΔ*hns*. Deletion of the *hns* gene resulted in poor growth in liquid culture compared to WT EHEC (Fig. S4A). Expression of T6SS-associated mRNAs was higher, although not as high as those found in t6sP::LA (Fig. 6A, see Fig. 1B). Once again, although we observed increased expression of *hcp3* and *tssB* mRNAs, Hcp3 protein expression was barely detectable by Western blot, and TssB was only found when using trans-expression with a pTrc plasmid (Fig. S4B). To rule out any problem with the antibodies, we designed a reporter system for t6sP, flanked by *gfp* and *mCherry*, representing the direction of the *tssB* and *hcp3* promoters, respectively. We found that *tssB* promoter expression was very low, as detected by GFP fluorescence in EHECΔ*hns* even after ON culture, whereas TssB expression could not be detected in WT EHEC under the same conditions (Fig. S4C). In the other direction, the *hcp3* promoter had a better expression of mCherry compared to almost null expression in WT EHEC. However, Hcp3 was not detected in the supernatant, suggesting poor activation of T6SS (data not shown). Together, this suggests that T6SS is poorly activated in EHECΔ*hns*, and perhaps further activation might be needed for T6SS function. To further understand this regulatory intergenic segment, reporter activity (mCherry) for h3R was measured in EHECΔ*hns* and compared to WT EHEC, but no difference was found in WT h3R or in mutants of regions I or I and II (Fig. S4D).

While little to no expression of T6SS proteins was found in LB, we tested other conditions to stimulate expression of T6SS proteins and found that Hcp3, TssB, and ClpV could be detected in EHECΔ*hns* (but not in WT EHEC) grown in DMEM or at lesser proportion in modified M9 (MM9: M9 + pyruvate and NaHCO_3_, used to improve T3SS protein secretion) (56). We could still not detect VgrG3 in EHECΔ*hns*, suggesting that the h3R is activated under different conditions (Fig. 6B). On the other hand, we searched for transcription factor binding sites for the t6sP regions to explain why MM9 and DMEM were better for protein expression and found several ArgP binding sites, as well as sites for CysB, CytR, Fis, LeuO, and NarP/NarL (Fig. S5). None of the sites could explain the increase in expression when induced with DMEM or MM9, and the fact that WT EHEC could not be activated in those media suggests that the activation is more complicated than simple binding of a transcription factor, although more studies are needed to ascertain the activation of this promoter.

When we analyzed the activity of EHEC secretion systems in EHECΔ*hns*, we found that T1SS and T2SS were over-secreting HlyA and StcE compared to WT EHEC. Interestingly, the T3SS substrates (Tir, EspA, and EspB) were not detected in EHECΔ*hns* but were detected in the WT EHEC, despite MM9 and DMEM being potent activators of injectosome activity. The EspP autotransporter (T5SS) also showed higher levels in EHECΔ*hns*, but only in DMEM. Hcp3 was also detected in the EHECΔ*hns* supernatant, but DnaK was also observed, which, together with the low growth, indicated cell lysis, probably due to phage activation (Fig. 6C). Indeed, Hcp3 could still be detected in the EHECΔ*hns*Δ*tssM* supernatant, confirming that the presence of Hcp3 is T6SS-independent (Fig. 6D). Our analysis determined that the *hns* mutation does not fully activate the T6SS, induces bacterial lysis, and dysregulates the secretion of important pathogenic factors in EHEC.

### T6SS regulation in EHEC depends on multiple signals and different promoters

**Fig 7:**
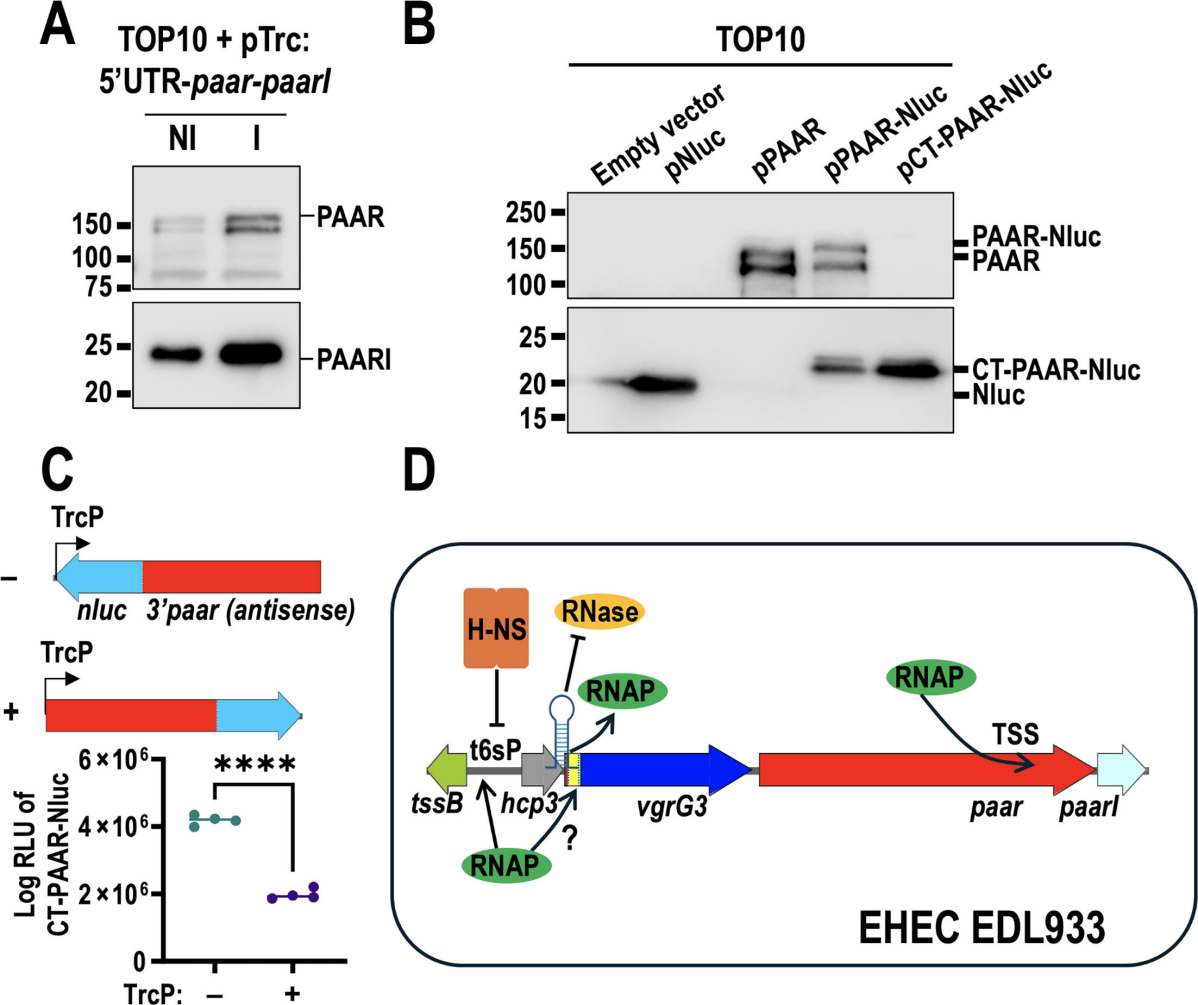
Dynamics of T6SS regulation in EHEC. (**A**) PAAR expression in TOP10 harboring a plasmid with the 5′ UTR of *paar*, along with the *paar* and *paarI* genes. (**B**) An additional transcription start site is located in the 3′ region of *paar*. (**C**) Transcriptional activity of 3′ *paar* TSS with (+) and without (–) a TrcP measured by luminescence. (**D**) Schematic representation of the regulation of the T6SS. RNAP represents the RNA polymerase. Region I of h3R is represented by a loop (blue) that protects *hcp3* mRNA from RNases. Promoter regions are required for RNAP to bind to the DNA to start transcription, although the accessory proteins that are required to displace H-NS and to bind to the DNA are still unknown. In addition, at least another transcription start site (TSS) in the 3′ region of *paar* may be influencing protein expression in EHEC. The 3′ region of *paar-nluc* (from +3187) was cloned out of frame from the native start present in pTrcHis2B, either in the sense of TrcP or in the antisense of the promoter. Luminescence was measured from ON culture, then the values of four biological replicates were plotted, and a Student’s *t*-test was performed (****; *P* < 0.0001). For protein expression, *E. coli* TOP10 was induced for 3 h, then protein was harvested, and 50 µg were separated by SDS-PAGE. Western blot was performed using polyclonal antibodies against PAAR or anti 6xHis tag for CT-PAAR-Nluc and PAARI.

Regulation of T6SS activity in EHEC appears to depend on multiple signals and different promoters, and mCherry/GFP fluorescence was too low in EHEC to be adequately studied. Thus, we changed to a bacterial luciferase reporter (*ilux*) where the promoters for *hcp3* or *tssB* were downstream of a TrcP. We tested several media to measure activation of *hcp3* or *tssB* promoters, according to the footprinting analysis we had performed previously (see Fig. S5) and using conditions that have been proven useful to activate T6SS-2 in *E. coli*. However, no condition was found to consistently activate any of the promoters, confirming our suspicions that regulation of T6SS does not depend on a single signal for activation (Fig. S6A and B). While h3R inhibits *vgrG3* expression *in vitro* and we did not find PAAR protein in WT EHEC, EHEC∆*hns,* or t6sP::LA, other studies suggest an additional transcription site inside *paar* (57). To determine whether the 5′ UTR region of *paar* inhibited PAAR expression and if there were additional transcription start sites inside the *paar* gene, we cloned the 5′ UTR along with *paar* and *paarI* under a Trc promoter. Both PAAR and PAARI were expressed, although the latter was more highly expressed, suggesting increased transcription or higher stability (Fig. 7A). Additionally, when we fused Nluc-6xHis (19 kDa) to the C-terminal of PAAR (157 kDa), we observed a protein of 177 kDa and an additional protein of approximately 23 kDa, which could constitute a small peptide (~3 kDa) fused with Nluc-6H (Fig. 7B), suggesting that protein translation initiates in the 3′ region of *paar*. To further demonstrate this, we cloned the 3′ region of *paar-nluc* using the internal HindIII site on a pTrc plasmid, on both the leading and complementary strands. In the Western blot assay, we observed the same protein at 23 kDa, larger than pNluc, suggesting that Nluc translation begins upstream in the 3*′ paar* region. Based on peptide size, we narrowed the possible start of translation to M1367 or V1382, the latter being more plausible as it generates a 23-amino-acid peptide (2.6 kDa), compared to 38 amino acids (for M1367), and possesses an RBS-like sequence (CGGTAAGGAC). When the 3′ *paar*-nluc was cloned in the complementary chain to TrcP, we observed higher expression by measuring luminescence activity (Fig. 7C), demonstrating that there is a native transcription and translation start site in the 3′ region of *paar*, since there are no other ORFs present in the complementary strand at pTrcHis2B, and a T1 terminator rrnB is located directly upstream of the cloned gene. Data from another group using dRNA-seq also indicates a transcription start site in the same region of *paar* (57). Along with the high production of PAARI, these results suggest that additional transcription start sites exist downstream of h3R, further complicating activation of T6SS.

## DISCUSSION

Three families of T6SS are commonly found in *E. coli*, of which T6SS-2 is the least studied, despite being associated with hypervirulent strains (26). A higher survival of EHEC inside macrophages due to the secreted catalase KatN (27) hints at the relevance of the T6SS in the pathogenesis of EHEC; however, it has not been deeply studied because it is not expressed *in vitro* conditions. Other studies have delved into the promoter activation, finding that *tssB* (*z0264*) was encoded as a different operon from *tssC*, suggesting that activation of different promoters was required to fully express T6SS structural proteins (40). Here, we find that promoter swapping was not enough for T6SS activation, as no substrate secretion, antibacterial activity, or Hcp3 translocation into HEp-2 cells was detected. While the proteins from the *tssB* operon were correctly expressed with a Tac promoter, in the other direction, the *hcp3* region activation only produced Hcp3, but not VgrG3 or PAAR, suggesting an additional control for the T6SS activity. By analyzing the intergenic region between *hcp3* and *vgrG3*, we found that those genes are encoded as different operons, thanks to a transcriptional terminator in the 5′ UTR of *hcp3*. As this region possessed a potential promoter region and several transcription factor-binding sites, we renamed it as h3R for *hcp3* regulatory sequence. We divided the h3R into three regions: region I contained a predicted transcriptional terminator; region II encoded a potential promoter region; and the third region corresponded to the 5′ UTR of *vgrG3*. Deletion of region I diminished *hcp3* mRNA, Hcp3 protein, and GFP reporter expression, while deletion of regions I and II was necessary for VgrG3 detection and increased mCherry reporter expression, whereas the presence of region III increased VgrG3 expression compared to its absence. Further, we demonstrate that although mRNA for T6SS genes is increased in EHECΔ*hns*, only Hcp3 was detected when bacteria were grown in LB, but ClpV and TssB were detected in *hns* mutants grown in DMEM and MM9, whereas VgrG and Rhs could not be detected. These data suggest that removal of H-NS is not sufficient for T6SS function, and specific stimuli must be needed for full activation of T6SS. We also provide additional information about the universal control of H-NS in other secretion systems, finding that all of them are affected by *hns* deletion. This work extends our knowledge on the activation of the T6SS and provides tools for studying T6SS expression and activation.

Promoter swapping was sufficient for Hcp secretion in *Citrobacter* (47), but was not enough to activate T6SS in *Shigella sonnei*, due in part to mutations in multiple T6SS core components (58). We found an increase in mRNA of the core components such as *tssB* after swapping promoters, although the leaky nature of the Tac promoter (59) translates into a high mRNA expression of *tssB* (Fig. 1B), as well as high expression of TssB and ClpV (whose gene is ten ORFs from *tssB*) even in non-induced conditions (Fig. 1C). Furthermore, promoter swapping was better for T6SS protein expression than using a *hns* mutant (see Fig. 6A). While Hcp3 was found in bacterial lysates, no VgrG3 or PAAR proteins could be detected either in the supernatant or in bacterial lysates (Fig. 1D; Fig. S1), while secretion of Tir, a T3SS substrate important for pathogenesis, was unaffected (Fig. S1). Furthermore, pedestal formation in the membrane of HEp-2 cells infected with t6sP::LA via T3SS confirmed that the promoter swapping did not affect a hallmark of EHEC pathogenesis (Fig. 2C).

We did not find antibacterial activity from WT EHEC or t6sP::LA (Fig. 2A and B), and previous bioinformatic analysis had not found obvious antibacterial effectors in EHEC genome, except perhaps RhsB, which has a RNase motif (17, 29). As the trans-expression of both VgrG3 and PAAR-PAARI in different plasmids was still not sufficient for substrate secretion nor antibacterial activity (Fig. S2A), additional mechanisms must be at play to hamper T6SS function. Lack of antibacterial activity could still be because T6SS-dependent antibacterial effectors are not being efficiently expressed *in vitro*, and although the structural proteins for the T6SS are being expressed, the assembly of the T6SS nanomachine could have been inhibited by unknown factors, thus inhibiting T6SS activity. EHEC has two orphan *vgrG* islands with Rhs effectors that were not dependent on the t6sP, as well as four orphan *rhs* genes that could possess antibacterial activity (29). Promoter swapping in EHEC Rafaela II or *E. coli* HS, which have different Rhs-PAAR proteins from EHEC EDL933 in the T6SS island, did not increase antibacterial activity, suggesting that these bacteria also have additional regulations preventing full activation. We also swapped the *vgrG1* promoter, and we did not find VgrG1 or RhsF, which could suggest that *vgrG1* is also being negatively regulated by an unknown post-transcriptional mechanism, as is the case for *P. aeruginosa*, where RsmA negatively regulates orphan *vgrG* genes (60).

The *hcp3* 3′ UTR is 520 bp downstream from the t6sP and contains, as we predicted, a transcription terminator, which divided the region into at least two operons encoding *hcp3* and *vgrG3*, respectively (Fig. 3A and B). Intrinsic transcription termination frequently includes the formation of a hairpin 80 bp downstream of the STOP codon (61), and we found a hairpin with 14 base-base interactions (Fig. 3C). We also found a promoter region upstream of *vgrG3*, which allowed us to rename the *hcp3* 3′ UTR as h3R and divide it into three regions: region I contained the predicted hairpin that could act as transcriptional terminator; region II encoded a potential promoter region with predicted binding sites for transcriptional factors; and region III corresponded to the 5′ UTR of *vgrG3*. Promoter swapping of the h3R for a Lac or an AraBAD promoter similarly did not increase VgrG3 expression to detectable levels in EHEC (data not shown), suggesting a strict mechanism for VgrG expression must be in place.

Interestingly, *hcp3* mRNA expression was significantly reduced after deletion of region I of the h3R (ΔI), suggesting that region I protects mRNA from RNases, although other mechanisms that change protein expression cannot be ruled out, such as polymerase backtracking (62). The predicted hairpin found in region I is probably responsible for transcriptional termination, as secondary structures have been associated with RNA pausing and termination (63). This hairpin could also protect *hcp3* mRNA, as hairpins can occlude recognition sites for ribonucleases or provide binding sites for RNA-binding proteins that protect from degradation (64, 65); thus, this could be a mechanism for region I to protect *hcp3* mRNA. Deletion of regions I and II increased VgrG3 expression and even changed *hcp3* and *vgrG3* from different operons to a single bicistronic operon (Fig. 4E), suggesting that the mechanism that decouples RNAP from DNA is present in these regions. This dual function of h3R to inhibit *vgrG3* and increase *hcp3* mRNA was conserved when replacing these genes for fluorescent reporters (Fig. 5B and C), confirming that the dual function of the h3R is only dependent on its sequence and not on the genes that are on either side of the regulatory sequence.

We found no VgrG3 in t6sP::LA bacteria, even though there was higher mRNA upon induction compared to WT EHEC (Fig. 1B through D), suggesting that a mechanism is impeding its translation post-transcriptionally. This kind of regulation has been reported for T6SS in *Pseudomonas*, where RsmA interacts with the 5′ UTR of mRNA for the T6SS genes (66). To alleviate repression, small non-coding RNA RsmZ and RsmY bind to RsmA, freeing the RBS sequence and permitting ribosome binding (67). Regulation of T6SS translation by other small RNAs has been reported for *V. cholerae* (68). Besides T6SS, other RNA-binding proteins have been found to regulate gene expression in EHEC. CsrA binds to the 5′ UTR region of *ehxB* and impedes its transcription, as well as accelerating its mRNA decay. CsrA also stabilizes *hlyE* by binding to its 5′ UTR region (69), shedding more light on the complex regulation of other secretion systems in EHEC.

Interestingly, it has been reported that genes *tssC* and *hcp3* from EHEC are being negatively regulated by CsrA (70). We did not find this effect on *tssB* nor in *vgrG3*, supporting our data about *hcp3* and *vgrG3* being two different operons. CsrA usually binds in the 5′ UTR at an AGGA/ANGGA motif, and we found such motifs upstream *tssC*, *tssB*, *hcp3*, and *paar*, at eight bases from the ATG, but not upstream *vgrG3*, although the complementary sequence was found 17 bp from the start codon. This evidence suggests that CsrA binding to the 5′ UTR region of *hcp3* mRNA leads to its degradation. However, it could also bind to the 3′ UTR and favor transcription termination, as has been reported for *pgaA* (71), thus inhibiting *vgrG3* termination, which would explain the increase in VgrG3 expression after deletion of regions I and II of the h3R. Therefore, further research is needed to pinpoint the real binding sites for CsrA and its role on T6SS regulation, which is further complicated because *csrA* deletion is lethal in *E. coli* (70).

The protein Hfq is also implicated in post-transcriptional regulation and in the regulation of T3SS in EHEC, although it plays opposite roles in different strains (72), as well as in the regulation of T6SS of *S. enterica* and *Pectobacterium carotovorum* (73, 74). However, there is no available information on the transcriptome changes after *hfq* deletion to pinpoint its role in T6SS activity, and further research is needed to uncover its implication in T6SS activity both in EHEC and related bacteria. Other small RNAs are responsible for Shiga toxin production (57), but to our knowledge, no such mechanism has been described for the T6SS of *E. coli*. While previous studies in *V. cholerae* have shown that *hcp* can be a separate operon from *vgrG* (75), no other studies have delved into the termination mechanism and the role of the intergenic region as a stabilizer of *hcp* that we are aware of.

While *hns* mutation has been shown to increase transcript levels of T6SS-associated genes (34), we demonstrate that it severely impacts bacterial growth (Fig. S4A), and mRNA transcription does not necessarily equate with protein expression, as TssB and ClpV were not detected in EHECΔ*hns*. Interestingly, EHECΔ*hns* induced in DMEM or MM9 increased T6SS-associated protein expression for some, but not all, proteins of the T6SS, suggesting that other proteins can bind to the t6sP and activate the translation of associated proteins (Fig. 6B). No T6SS-related proteins were detected in WT EHEC grown in DMEM, suggesting that H-NS must be released from DNA before other proteins could bind. DMEM has been shown to activate T6SS-3 in EAEC 17-2 by the AggR pathway, while Ler is also activated in EHEC and EPEC grown in DMEM, leading to the activation of the T3SS injectosome (76). Importantly, we observed no secretion of T3SS substrates upon deletion of *hns* (Fig. 6C), and we did not find T3SS EspA in bacterial lysates either (data not shown), suggesting a total inhibition of the T3SS. Although Ler is known to counteract the effect of H-NS-mediated repression of T3SS in EPEC (54), a study of EHECΔ*hns* shows an important decrease in T3SS-associated genes upon deletion of *hns* (34), suggesting that the regulation of T3SS in EHEC is different from EPEC. Indeed, we have observed that T3SS activity in DMEM is lower in EHEC than EPEC (data not shown), indicating that additional mechanisms are to be at play alongside Ler and H-NS, and the regulation of T3SS has been shown to depend on many factors beyond the Ler-H-NS pathway (77).

Even though EHECΔ*hns* expressed some proteins of the T6SS when activated in DMEM, VgrG3 and Rhs proteins were still absent in total lysates, suggesting that an additional stimulus must be needed for full activation. While *vgrG3* mRNA was upregulated in EHECΔ*hns* (Fig. 6A), no change in expression was detected using fluorescent reporters (Fig. S4D), suggesting an additional post-transcriptional mechanism of *vgrG3* regulation. Although Hcp3 was detected in the supernatant, we confirmed that the presence was due to bacterial lysis instead of T6SS activation (Fig. 6D). We have observed that StxA secretion is higher in EHECΔ*hns* (data not shown) and, along with the poor fitness of the *hns* mutant (Fig. S4A), suggests bacterial lysis due to phage activation. Deletion of *hns* remains a powerful tool for functional analysis and is very useful for the study of bacterial epigenetics, but our findings highlight the need for an alternative to study secretion in EHEC, as all the secretory systems we analyzed were altered in this mutant.

The 5′ UTR region of *paar* had no identifiable hairpins, and trans-expression of PAAR was achieved when cloned in a plasmid with its 5′ UTR, suggesting that no mechanisms inhibiting transcription or translation are present there (Fig. 7A). Interestingly, we found higher levels of PAARI than PAAR, which is encoded downstream (Fig. 7D). Analyses of dRNA-seq and RNA-seq from other groups have confirmed that mRNA expression in the 3′ region of *paar* is higher than in the 5′ UTR (34, 57). We detected a small Nluc-bound PAAR peptide upon trans-expressing the 3*′ paar* region in EHEC (~3 kDa plus Nluc, Fig. 7B and C). The presence of the ~23 kDa protein in the absence of the full gene confirms that this is not a product of cleavage as in other Rhs proteins (78), but represents its own ORF, with a transcription start site, as revealed by luminescence assays (Fig. 7C). We propose that translation initiates at V1382, producing a small 23-amino-acid peptide (2.6 kDa) that has an RBS-like sequence directly upstream. Taken together, these results indicate that resolving *vgrG3* inhibition is not enough for T6SS activity and additional control mechanisms exist to prevent non-intended activation.

Several mutations inside the T6SS pathogenic island could suggest that T6SS is not functional in EHEC. Mutations in *z0246-z2045* divide a potential antibacterial effector into two presumably non-functional ORFs, similar to *tssA*, which is divided into two ORFs due to a nonsense mutation. Mutations of core genes have been shown to produce functional proteins where both the truncated and the full version are necessary for T6SS function (79). Another study that tried promoter swapping observed no T6SS-dependent antibacterial activity, and further investigation revealed mutations of core components leading to a loss of T6SS function (58). However, such mutations are not present in *E. coli* str. HS, and protein secretion was not achieved even after promoter swapping or *hns* mutation, bringing more evidence to our hypothesis that additional stimuli are needed for full T6SS activation. Similarly, although T6SS function is usually characterized as antibacterial or anti-eukaryotic, an additional function for the T6SS cannot be ruled out. Recent studies associate *clpV* with STEC found in humans, suggesting that T6SS is somehow involved in human pathogenesis (28). The EHEC str. zh165 is known to possess antibacterial activity due to an Rhs protein with an RNase domain (17), but the complete sequence of the T6SS locus is not available, making it impossible to compare the *tssM* and *tssA* genes. It has also been reported that EHEC O157:H7 is outcompeted by other STEC strains, such as serotype O22:H8, which prevents O157:H7 infection in cattle and directly kills O157:H7 *in vitro* (80, 81).

Although this work sheds light on the mechanisms that regulate T6SS expression, the natural signals that activate the T6SS remain unknown, and further research is needed to pinpoint the exact transcription factors that play a role in the activation of the T6SS island and of the orphan effectors encoded elsewhere in the chromosome. In addition to the regulatory mechanisms found in this work, other post-transcriptional or post-translational mechanisms could be preventing the expression, assembly, or activation of T6SS, so further work will be needed to fully understand the regulatory pathways of T6SS in EHEC and other bacteria.

In conclusion, we demonstrated that regulation of T6SS in EHEC is not dependent only on the t6sP, as protein expression using promoter swapping is not sufficient for Hcp secretion. We also characterize a regulatory sequence between *hcp3* and *vgrG3*, which we divided into three regions containing a transcriptional terminator, a promoter region, and the 5′ UTR of *vgrG3*. As the T6SS is a machinery that helps pathogenic bacteria infect a host due to their anti-bacterial or anti-eukaryotic activities, understanding the underlying mechanisms of T6SS activation helps us understand the precise sequence of events that trigger infection (Fig. 7D). EHEC is commonly found in the microbiome of healthy people, and EHEC infection can vary from mild diarrhea to lethal HUS, and the fine-tuning of its many pathogenicity factors must play an important role in the severity of the disease.

## SUPPLEMENTAL MATERIAL

Supplemental tables spectrum.03548-25-s0001.docx Tables S1 and S2.

Supplemental material spectrum.03548-25-s0002.docx Supplemental figure legends.

Fig. S1 spectrum.03548-25-s0003.tif No secretion of substrates was detected after promoter swapping.

Fig. S2 spectrum.03548-25-s0004.tif Promoter swapping did not result in antibacterial activity nor protein translocation to HEp-2 cells.

Fig. S3 spectrum.03548-25-s0005.tif Reporter expression for h3R under different media.

Fig. S4 spectrum.03548-25-s0006.tif Transcription factor binding site prediction in the t6sP of EHEC EDL933.

Fig. S5 spectrum.03548-25-s0007.tif Deletion of the hns gene negatively impacts growth and only modestly affects expression of T6SS-related proteins.

Fig. S6 spectrum.03548-25-s0008.tif Analysis of t6sP in different media using ilux.

ASM does not own the copyrights to Supplemental Material that may be linked to, or accessed through, an article. The authors have granted ASM a non-exclusive, world-wide license to publish the Supplemental Material files. Please contact the corresponding author directly for reuse.
