# Supplementary material for: An hcp3-vgrG3 intergenic region participates in EHEC T6SS expression in addition to the bidirectional promoter and H-NS
Source: Microbiol Spectr. 2026 Apr 14;14(5):e03548-25. doi: 10.1128/spectrum.03548-25 (PMC13141884; doi:10.1128/spectrum.03548-25)
Supplement: Supplemental tables — Tables S1 and S2. [file spectrum.03548-25-s0001.docx]

**Table S1. Strains and plasmids used in this study**

| **Strain or Plasmid** | **Description** | **Reference** |
| --- | --- | --- |
| ***Strains*** | | |
| EHEC EDL933 | Prototype isolated EHEC O157:H7 (Wild-type) | Gifted by Puente-Garcia, JL. |
| EHECΔ*hns* | EDL933::*hns::kanR* | This study |
| EHECΔ*tssM* | EDL933::*tssM::kanR* | This study |
| EHECΔ*hns*Δ*tssM* | EDL933::*hns::kanR::tssM::cmR* | This study |
| t6sP::LA | T6SS promoter swapped by AraBAD and TacP | This study |
| t6sP::AL | T6SS promoter swapped by TacP and AraBAD | This study |
| t6sP::LAΔ*tssM* | EHEC::t6sP::Lac-Ara::*tssM*::kanR | This study |
| t6sP::LAΔ*clpV* | EHEC::t6sP::Lac-Ara::*clpV*::kanR | This study |
|  |  |  |
| BL21(DE3) | Strain used for protein expression | Invitrogen |
| TOP10 | Plasmid storage and gene cloning | Invitrogen |
| EHEC str. Rafaela II | EHEC O157:H7 str Rafaela II | Gift from Cataldi, AA. |
| EAEC str 042 | Model for enteroaggregative *E. coli* |  |
| EASTEC O104:H4 | EAEC with *stx*, serotype O104:H4 | Gift from Farfan, MJ. |
| **Plasmids** | | |
| pKD4 | R6K plasmid with kanR flanked by FRT sites. | (1) |
| pKD46 | For recombineering using lambda red recombinase | (1) |
| pRL128 | For promoter swapping | (2) |
| pFCcGi | vector with *gfp* and *mCherry* | (3) |
| *ilux* pGEX | *ilux* on a Tac promoter | (4) |
| pGFP-Nluc | pRetroX-Tight-MCS_PGK-GpNLuc | (5) |
| pTrcHis2B | TrcP, AmpR, CT-6xHis, for protein expression | Invitrogen |
| pRSET A | T7P, AmpR, NT-6xHis, for protein expression | Invitrogen |
| pTrc-4C6H | 4xCys added between EcoRI and HindIII of pTrcHis2B | This study |
| pTrc:Hcp3-4C6H | *hcp3* cloned in pTrc-4C6H (NcoI, BglII) | This study |
| pTrc:Hcp3 | *hcp3* without 6 x His, cloned in pTrc-4C6H (NcoI, XhoI) | This study |
| pTrc:VgrG3 | *vgrG3* cloned in pTrc-4C6H (NcoI, PstI) | This study |
| pTrc:PAAR | *paar* cloned in pTrc-4C6H (NcoI, PstI) | This study |
| pTrc:TssB | *tssB* cloned in pTrcHis2B (XhoI, KpnI) | This study |
| pTrc:Hcp3-h3R-VgrG3 | *hcp3-vgrG3* region cloned in pTrc-4C6H | This study |
| pTrc:Hcp3-h3RΔI-VgrG3 | *hcp3-vgrG3* region with deletion of 31 bp, cloned in pTrc-4C6H | This study |
| pTrc:Hcp3-h3RΔI-VgrG3 | *hcp3-vgrG3* region with deletion of 170 bp, cloned in pTrc-4C6H | This study |
| pTrc:h3R-VgrG3 | *h3R* region and *vgrG3* cloned in pTrc-4C6H | This study |
| pTrc:h3RΔI-VgrG3 | *h3R* region minus 39 bp and *vgrG3* cloned in pTrc-4C6H | This study |
| pTrc:h3RΔIΔII-VgrG3 | h3R minus 170 bp and *vgrG3* cloned in pTrc-4C6H | This study |
| pTrc:GFP-h3R-mCherry | *h3R* region flanked by *gfp* and *mCherry* | This study |
| pTrc:GFP-h3RΔI-mCherry | h3R region flanked by *gfp* and mCherry, with deletion of 31 bp | This study |
| pTrc:GFP-h3RΔIΔII-mCherry | h3R region flanked by *gfp* and mCherry, with deletion of 170 bp | This study |
| pTrc:Hcp3-h3R-mCherry | *h3R* region flanked by *hcp3* and *mCherry* | This study |
| pTssB-VgrG3 | *tssB-vgrG3* region, including t6sP and h3R, cloned in pFCcGI between KpnI and PstI | This study |
| pGEX:hcp3P:*ilux* | Promoter region for *hcp3* cloned upstream *ilux* operon | This study |
| pGEX:tssBP:*ilux* | Promoter region for *tssB* cloned upstream *ilux* operon | This study |
| pTrc:*hcp3*-h3R-*vgrG3*-:*ilux* | *hcp3-h3R-vgrG3* cloned upstream *ilux* operon |  |
| pTrc:ClpV-GFP | 5’ UTR of *clpV* and ClpV fused with GFP in the C-terminal with a Trc promoter | This study |
| p15A-Trc | pTrc-4C6H with p15A ori | This study |
| pTrc:VgrG3-Nluc | VgrG3 fused to Nluc in the C-terminal, under a Trc promoter and a p15A ori | This study |
| pTrc:PAAR-Nluc | PAAR fused to Nluc in the C-terminal, under a Trc promoter | This study |
| pTrc:5’UTR-PAAR-PAARI | 5’UTR region from *paar*, along with *paar* and *paarI* under a Trc promoter | This study |
| pTrc:3’*paar*-Nluc-6H (sense) | 3’ region of *paar-nluc* cut from pTrc:PAAR-Nluc with HindIII and SalI, then cloned in pTrcHis2B, so it was downstream of TrcP. | This study |
| pTrc:3’*paar*-Nluc-6H (Antisense) | 3’ region of *paar-nluc* cut from pTrc:PAAR-Nluc with HindIII and SalI, then cloned in pTrcHis2B using HindIII and XhoI, so the *paar-nluc* region was in the complementary chain as the TrcP. | This study |

**Table S2. Primers used in this study**

| **Name** | **Sequence (5’-3’)** |
| --- | --- |
| ***Gene deletion*** | |
| clpV-KO-F | ACGCAGGACATCCTGCACCAGCTAAACAAATTACTTCGATAAGGACGTTTTGTGTAGGCTGGAGCTGCTT |
| clpV-KO-R | CCACTGCCGACAACCGAATCCGGGCAATCGCCGTGTTAGCCGCCATTCATATGAATATCCTCCTTAG |
| tssM-KO-F | TGCCCCGGTTTAAAGTCTCCGCTTTCTGGCTGCTGATACTGGCGTGGATTTTTCTGCTTGTGTGTAGGCTGGAGCTGCTT |
| tssM-KO-R | GGGTCACTGCCGGTAACGATAGTTTGTGTCAGTACGTTACTATTCATCCCTGCCTCCTCACATATGAATATCCTCCTTAG |
| hns-KO-F | TCTATTATTACCTCAACAAACCACCCCAATATAAGTTTGAGATTACTACAGTGTAGGCTGGAGCTGCTTC |
| hns-KO-R | TAAAAAATCCCGCCGATGGCGGGATTTTAAGCAAGTGCAATCTACAAAAGACATATGAATATCCTCCTTAGTTC |
| ***Gene cloning*** | |
| 4CysF | AATTGGCTGGATCCTTTCTTAATTGCTGCCCCGGGTGCTGCATGGAGCCCGAATTCGGCGGCCGCA |
| 4CysR | AGCTTGCGGCCGCCGAATTCGGGCTCCATGCAGCACCCGGGGCAGCAATTAAGAAAGGATCCAGCC |
| hcp3-NcoI-F | CATGCCATGGGGATGCCAACACCGTGTTATATC |
| hcp3-BglII-R | GAAGATCTTGCTTCCAGCGGTGCGCGC |
| hcp3-XhoI-R | CCGCTCGAGTTATGCTTCCAGCGGTGCGC |
| vgrGU-NcoI-F | CATGCCATGGCCATGTCAACCGGATTACGTTTCAC |
| vgrG3-PstI-R | TGCACTGCAGGAAATTGCCGCATATCCGGCATTC |
| 5’-clpV-F | GCTGGTCCATTTCCACAACG |
| *clpV*-NcoI-R | CATGCCATGGCTAAAACCGTTTCATCCTTTGTG |
| h3R-XhoI-F | TCACTCGAGTTAAGCCAACAGCATCCGGCT |
| h3RΔI-XhoI-F | GTCACTCGAGATGCTGTTTTTGTATTACGCCGTAG |
| h3RΔIΔII-XhoI-F | ACACTCGAGACAGCGAACTGTCTGTTTTTCC |
| gfp-BsaI-F | CAGGTCTCACATGAGTAAAGGAGAAGAACT |
| gfp-XhoI-R | ACTGCTCGAGTTATTTGTATAGTTCATCCATGCCATGTG |
| mCherry-BsaI-F | CAGGTCTCATGGTGAGCAAGGGCGAGGA |
| mCherry-HindIII-R | CATTAAAGCTTTCGCGGCCGC |
| tssBXhoIF | CCGCTCGAGCATGAGCAAAAAATTTGAAGG |
| tssBKpnIR | CGGGGTACCGTTTATTGCTCACCTGGC |
| pTrc-CmR-p15AF | TGACGGATGGCCTTTTTGCGGGTCGAATTTGCTTTCGAA |
| pTrc-CmR-p15AR | CGGTGATGACGGTGAAAACCTGATTAATAAGATGATCTTCTTGAGATCG |
| NlucPstIF | AGATCTGCAGTCTTCACACTCGAAGATTTCG |
| NlucSalIR | GATGGTCGACCGCCAGAATGCGTTCGC |
| PAARNcoIF | GACACCATGGGAGGAAAACCGGCGGC |
| PAARPstIR | TGCACTGCAGGTTTGCTTCCTGTAGGTGTAAACTTATGAAAGTC |
| 5paarNcoIF | CTCACCATGGCGGCAATTTTGAGTCAGGTG |
| paarISalIR | CGACGTCGACCTTTTGTTCTTTCGTTAATTCTTCAATTAGTCGATCTGC |
| 5-iluxNotIF | AATGCGGCCGCACACAGGAAACAGGATCCA |
| ilux-NotIR | ACGATGCGGCCGCTTACCTTCT |
| t6sPBamHIF | ACAGGATCCGATCCGTCATGTTATCTCGT |
| t6sPBamHIR | ACAGGATCCACTCCTTGTTAACGTGGTTA |
| ***RT-PCR*** |  |
| rna16S-EDL933-F | AAGACCAAAGAGGGGGACCT |
| rna16S-EDL933-R | TGTCTCAGTTCCAGTGTGGC |
| tssB-RT-F | GAACGTCAGGCAGTTTCCGT |
| tssB-RT-R | GGCCACGCTATCTGGTGAAA |
| hcp3-RT-F | ACGTTACTGTCCCGACCGAT |
| hcp3-RT-R | GCTCCTGTTTGCCTTCCACA |
| vgrG3-RT-F | CGGCACGATATTGCAGGAAA |
| vgrG3-RT-R | CCGGCAGATAAAGCACGGTA |
| ***Promoter swapping*** | |
| t6sP-tacP-P2-F | CGAGGCGCGACGCTGCCTTCAAATTTTTTGCTCATAAAAGTGATCCGTCATGTTATCTCGTACAGTTGGCTGTTTCCTGTGTGAAATTG |
| t6sP-araBAD-P1-R | CTGACCAGTGATGGAGATATAACACGGTGTTGGCATTTGTAAACTCCTTGTTAACGTGGTTAATTACGGGTATGGAGAAACAGTAGAGAGTTG |
| V3pP4lacF | CACGTCAAATCTGGCACCTCCGGTGCCGACGACTGGCGCGCACCGCTGGAAGCATAAGTTAAGCCAACGacctgcagttcgaagttcc |
| V3pP2Lac-20R | CGGTGGCAGGCCGTCCACTTCCAGCGTGAAACGTAATCCGGTTGACATAGCAACCTCCGGGGTTAAGGtTGGCTGTTTCCTGTGTGAAATTG |
| t6sP-araBAD-P2-F | GATATAACACGGTGTTGGCATTTGTAAACTCCTTGTTAACGTGGTTAATTACTTGGCTGTTTCCTGTGTGAAATTG |
| t6sP-tacP-P1-R | GCCTTCAAATTTTTTGCTCATAAAAGTGATCCGTCATGTTATCTCGTACAGGGGTATGGAGAAACAGTAGAGAGTTG |

1. Datsenko KA, Wanner BL. 2000. One-step inactivation of chromosomal genes in *Escherichia coli* K-12 using PCR products. Proceedings of the National Academy of Sciences 97:6640-6645.

2. Gueguen E, Cascales E. 2013. Promoter swapping unveils the role of the *Citrobacter rodentium* CTS1 type VI secretion system in interbacterial competition. Appl Environ Microbiol 79:32-38.

3. Figueira R, Watson KG, Holden DW, Helaine S. 2013. Identification of *Salmonella* pathogenicity island-2 type III secretion system effectors involved in intramacrophage replication of *S. enterica* serovar typhimurium: implications for rational vaccine design. MBio 4:10.1128/mbio. 00065-13.

4. Gregor C, Gwosch KC, Sahl SJ, Hell SW. 2018. Strongly enhanced bacterial bioluminescence with the ilux operon for single-cell imaging. Proceedings of the National Academy of Sciences 115:962-967.

5. Sibinelli-Sousa S, Hespanhol JT, Nicastro GG, Matsuyama BY, Mesnage S, Patel A, de Souza RF, Guzzo CR, Bayer-Santos E. 2020. A Family of T6SS Antibacterial Effectors Related to l, d-Transpeptidases Targets the Peptidoglycan. Cell Reports 31:107813.
