# Supplementary material for: An hcp3-vgrG3 intergenic region participates in EHEC T6SS expression in addition to the bidirectional promoter and H-NS
Source: Microbiol Spectr. 2026 Apr 14;14(5):e03548-25. doi: 10.1128/spectrum.03548-25 (PMC13141884; doi:10.1128/spectrum.03548-25)
Supplement: Supplemental material — Supplemental figure legends. [file spectrum.03548-25-s0002.docx]

**Supplemental Figures**

**Fig. S1. No secretion of substrates was detected after promoter swapping. (A)** Secretion of T6SS substrates to the supernatant by t6sP::LA. **(B)** Hcp3 secretion using FlAsH **(C)** VgrG3 and PAAR secretion measured with luminescence. **(D)** Comparison of T6SS protein expression after promoter swapping using. In t6sP::LA *tssB* operon is under Lac promoter. In t6sP::AL *hcp3* is under control of Lac promoter. Bacteria were induced 3 h with 0.2 mM IPTG and/or 0.2% arabinose, then RNA was obtained with TRIzol, while proteins from the supernatants were precipitated with Trichloroacetic acid (TCA) at a final concentration of 15%. The equivalent of 3 ml was analyzed by Western blot and T3SS-Tir was used as a positive control for protein secretion. For FlAsH secretion, bacteria containing a plasmid that encoded Hcp3 with a 4-Cys tag were incubated with LUMIO™ 0.2 µM and the fluorescence was measured in the supernatant using a microplate Spectrophotometer. Welch's t-test was used for comparison. (ns, not significative). For luminescence analysis, plasmids containing VgrG3 or PAAR proteins fused with a Nluc in the C-terminus were used and Furimazine was added as substrate of the Nluc. One way ANOVA followed by a Šídák's multiple comparisons test was used to estimate significant differences in the samples (ns, not significative).

**Fig. S2. Promoter swapping did not result in antibacterial activity nor protein translocation to HEp-2 cells. (A)** Prey CFUs recovered in presence of different predators. **(B)** Comparison between t6sP and h3R from EHEC EDL933 agains other *E. coli*. **(C)** Detection of Hcp3 and VgrG3 in the cytosol or the membranes fraction of HeLa cells infected with WT EHEC, t6sP::LA (LA) or t6sP::LAΔ*tssM*. For competition assays, Bacteria were grown in LB at 37°C until an OD of 0.8-0.9, then adjusted to 0.5 and mixed in a 4:1 ratio (predator:prey). Samples of 20 µL were spotted into LB plates supplemented with 0.2% arabinose and 0.1 mM IPTG and incubated ON at 30°C. Spots were then resuspended in PBS and serially diluted to obtain countable colonies. Rafaela::LA is a Rafaela II strain with promoter swapping t6sP::Lac-Ara. Rafaela::AL is a Rafaela II strain with promoter swapping t6sP::Ara-Lac. HS::LA is a *E. coli* str HS strain with promoter swapping t6sP::Lac-Ara. A One-Way ANOVA test was performed followed by a Šídák's multiple comparisons test (*, P <0.05). For B, Nucleotide BLAST was performed for the region between *hcp3* and *tssB* (t6sP) and for the region between *hcp3* and *vgrG3* (h3R). For infection assays, EHEC and its variants were induced with 0.2 mM IPTG and 0.2% arabinose in MM9 for 4 h, then bacterial cells were washed and resuspended in DMEM. HeLa cells at 70% confluence were fasted ON and then infected with a MOI of 10 for 4 h. After infection, proteins from cytoplasm and membrane fractions were separated and separated by SDS-PAGE (50 µg). Anti-GAPDH and anti-Calnexin were used to evaluate the purity of cytoplasmic and membrane fractions, respectively.

**Fig. S3. Reporter expression for h3R under different media.** Schematic representation of mCherry cloned with WT h3R, deletion in region I, region I&II or directly cloned under Trc promoter (ΔIΔIIΔIII). **(B)** Fluorescence of mCherry from plasmids in A. Overnight cultures were resuspended 1:10 in fresh media with 0.2 mM IPTG, then incubated at 37°C for 6 h, measuring fluorescence each 30 min. **(C)** h3R reporter fluorescence after 6 h of induction in different conditions. For IPTG condition, 0.2 mM and 0.2% glucose were added. For Arginine, Alanine, Aspartate or Asparagine 0.5 mM were added, as well as 0.2% glucose. For Glucose, Arabinose, Melibiose, Stachyose, Mannose, Lactose, Galactose or Xylose, 0.2% were added. One-Way ANOVA with Dunnett's multiple comparisons test was used to compare different media (***, P <0.001).

**Fig. S4. Transcription factor binding site prediction in the t6sP of EHEC EDL933. (A)** Map of the T6SS pathogenicity island. **(B)** Virtual footprint for the whole t6sP. **(C)** Close up of the -35 box.

**Fig. S5. Deletion of *hns* gene negatively impacts growth and only modestly affects expression of T6SS related proteins. (A)** Growth kinetics for EHEC EDL933 WT and *hns* mutant **(B)** Expression of Hcp3, TssB and VgrG3 in EHEC and EHECΔ*hns* using a trans-expression vector containing genes *tssB*, *hcp3* and *vgrG3*, along with their respective promoters. **(C)** Reporter assembly to measure t6sP expression in EHEC and EHECΔ*hns* in ON culture. **(D)** Reporter expression for h3R and its mutants were unaffected by *hns* mutation. WT EHEC or EHECΔ*hns* were grown from ON cultures diluted 1:10 for 6 h at 37°C, and OD 600 nm was measured every 30 min, as well as mCherry fluorescence for D. Data was obtained from at least three independent experiments. RNA expression was analyzed using Two-Way ANOVA followed by a Šidák multiple comparisons test. (**, P <0.01; ****, P <0.0001). Proteins were separated by SDS-PAGE and analyzed by Western blot to detect T6SS proteins. DnaK was used as a loading control.

**Fig. S6. Analysis of t6sP in different media using *ilux*. (A)** Bioluminescence for *tssB* promoter in different media. **(B)** Bioluminescence for *hcp3* promoter in different media. Overnight cultures were diluted 1:25 and incubated 6 h at 37°C. For Asparagine (Asn) and Arginine (Arg), 0.5 mM was added, as well as 0.2% glucose. For Arabinose (Ara), Glucose (Glc), Xylose (Xyl), Galactose (Gal) and Mannose (Man) or, 0.2% was added. Other concentrations were 500 nM of ZnSO_4_ and CuSO_4_; 6 mM H_2_O_2_. Modified M9 (MM9), M9, Sci-I inducing medium (SIM) or Tryptic Soy Broth (TSB) were used. One-Way ANOVA with Dunnett's multiple comparisons test was used to compare different media, and only significative comparisons with means higher than LB were drawn (****, P <0.0001).
