# Supplementary figures and images for: An hcp3-vgrG3 intergenic region participates in EHEC T6SS expression in addition to the bidirectional promoter and H-NS
Source: Microbiol Spectr. 2026 Apr 14;14(5):e03548-25. doi: 10.1128/spectrum.03548-25 (PMC13141884; doi:10.1128/spectrum.03548-25)

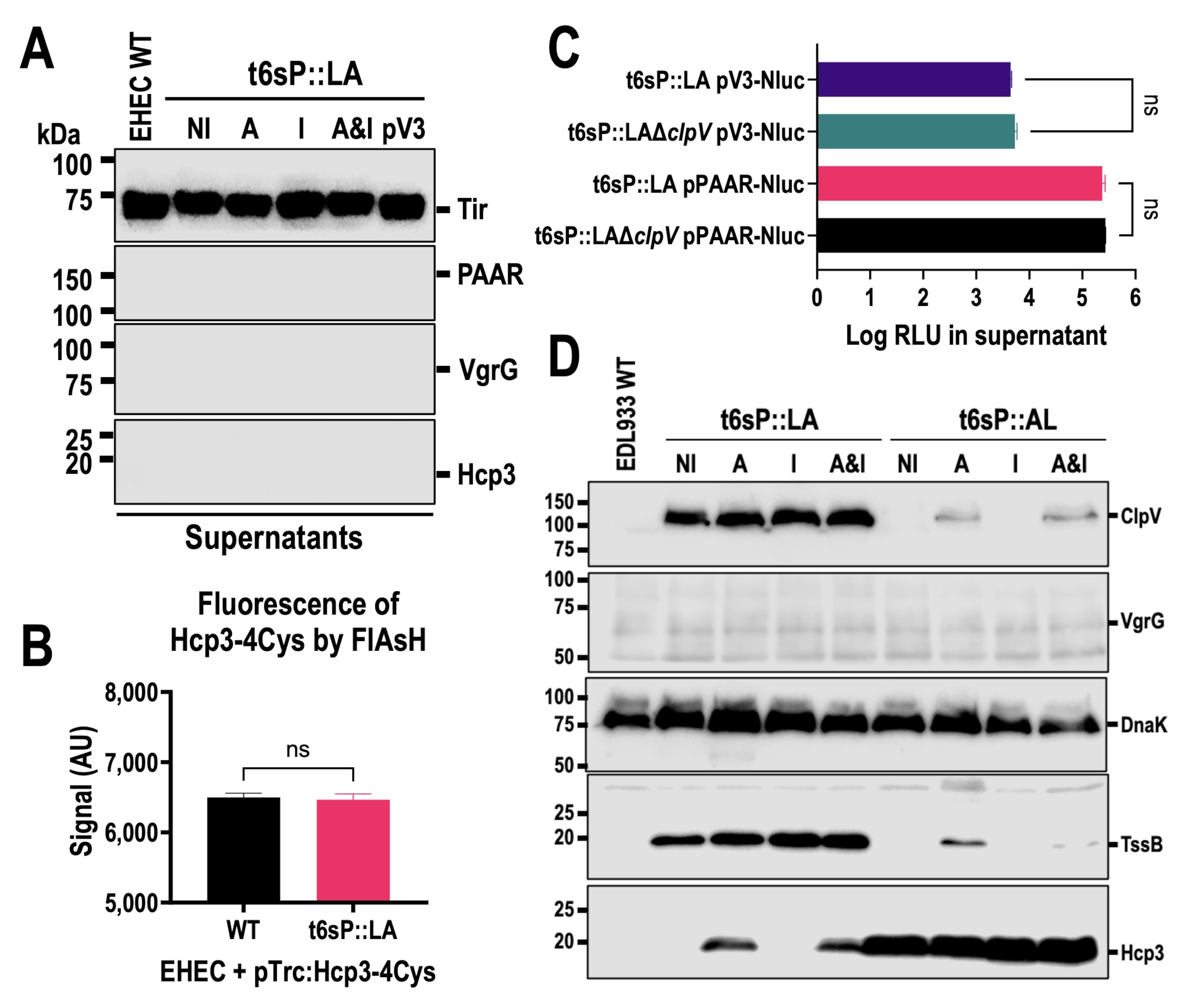

Supplement: Fig. S1 — No secretion of substrates was detected after promoter swapping. [file spectrum.03548-25-s0003.tif]

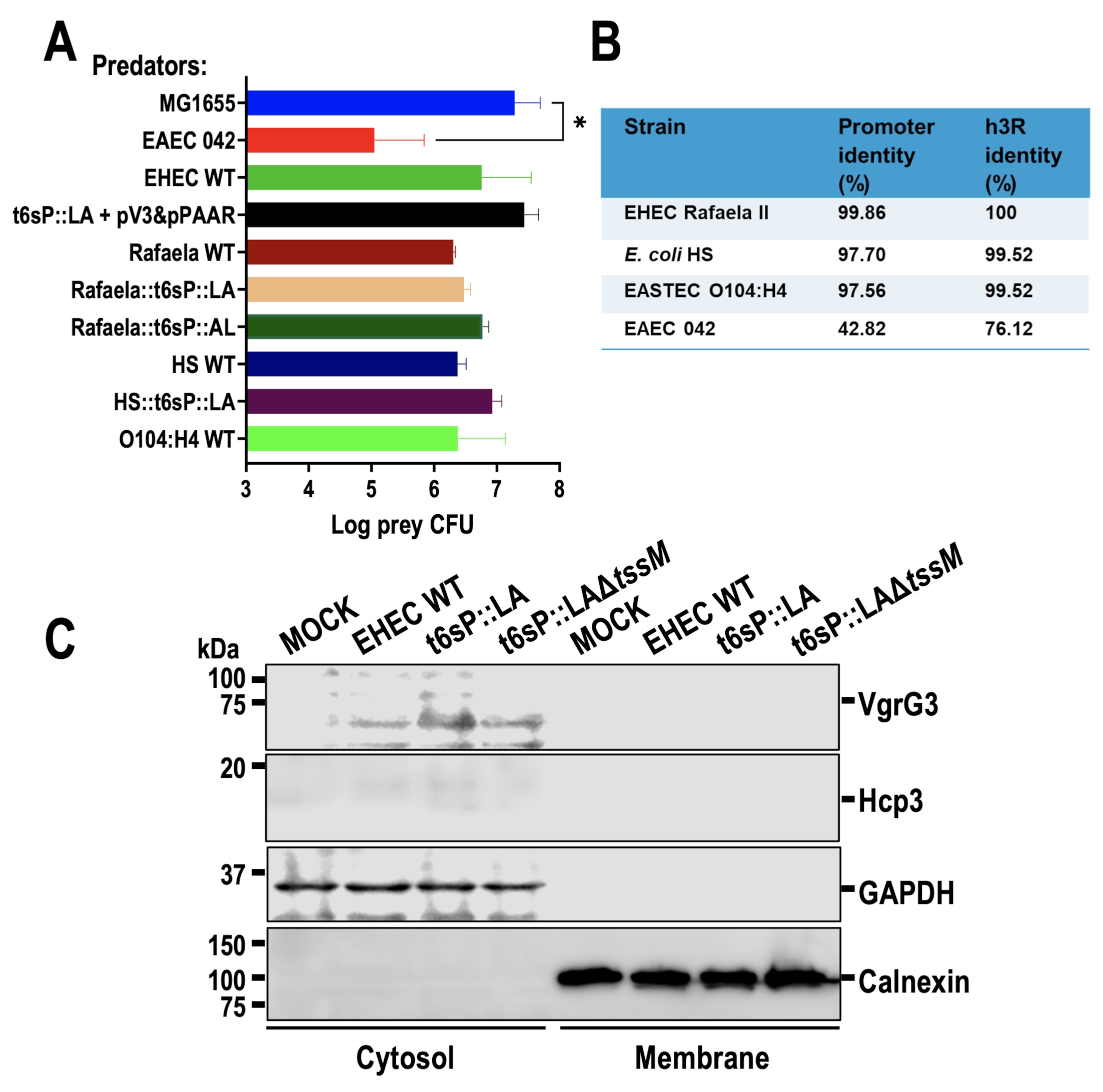

Supplement: Fig. S2 — Promoter swapping did not result in antibacterial activity nor protein translocation to HEp-2 cells. [file spectrum.03548-25-s0004.tif]

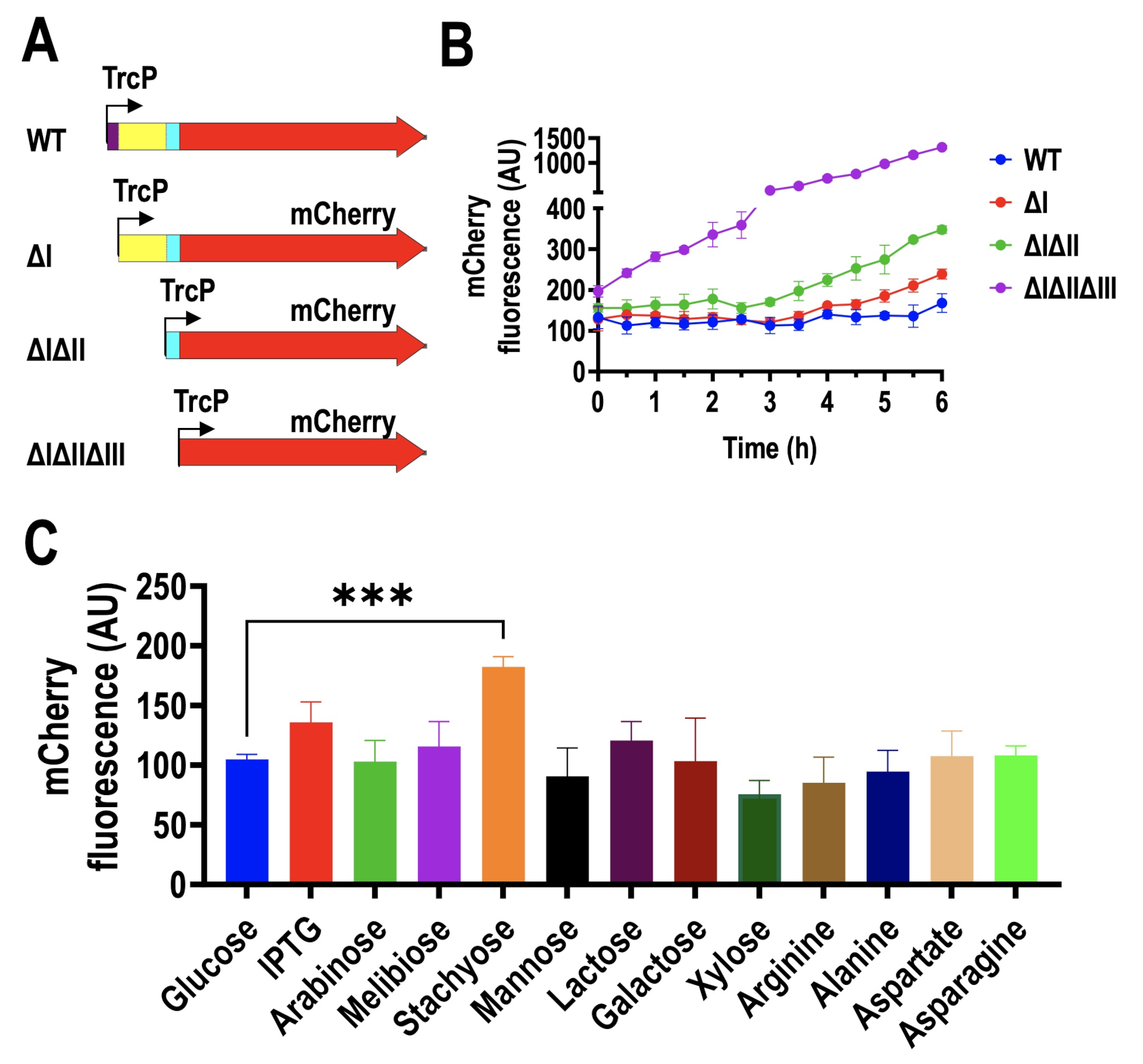

Supplement: Fig. S3 — Reporter expression for h3R under different media. [file spectrum.03548-25-s0005.tif]

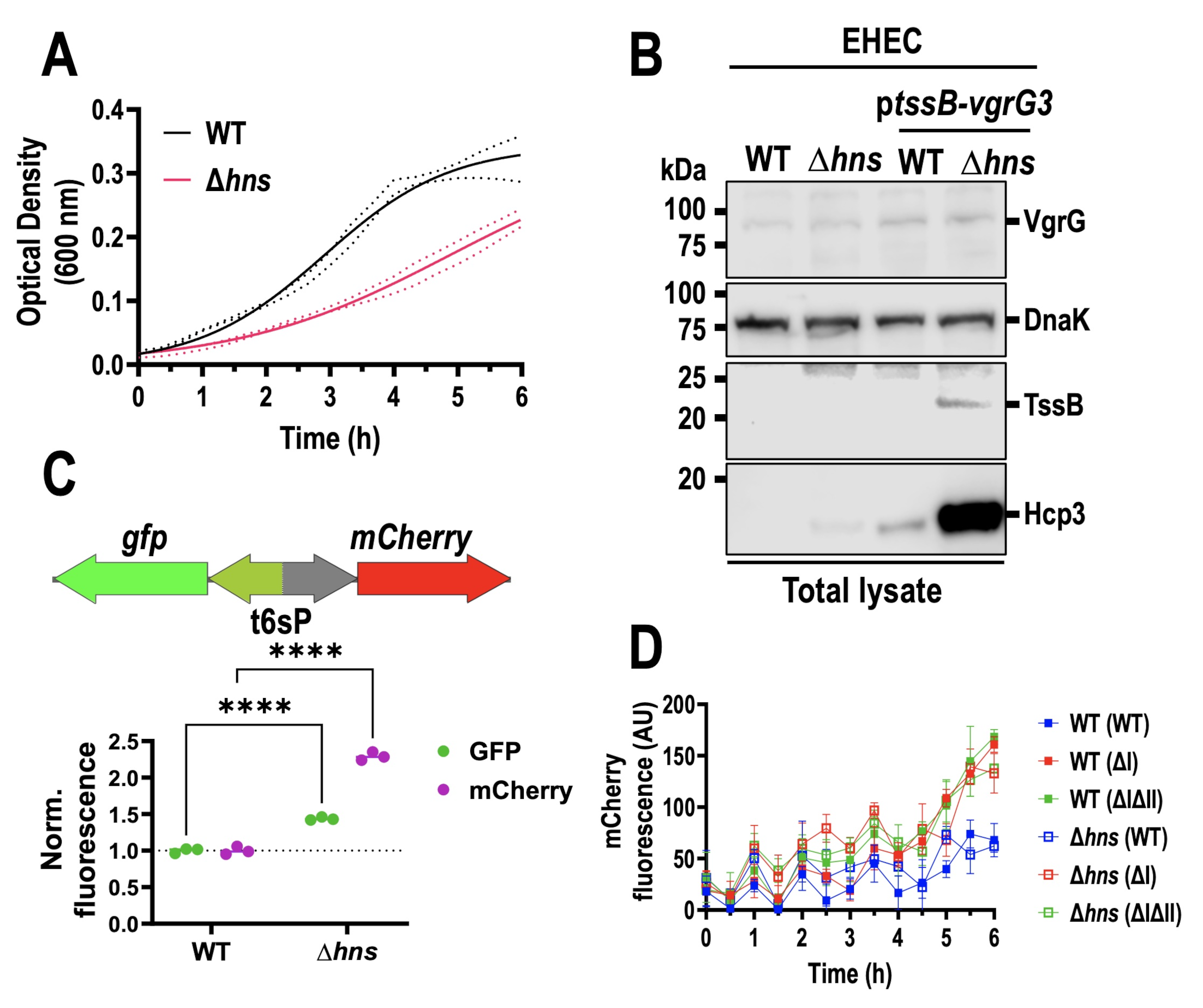

Supplement: Fig. S4 — Transcription factor binding site prediction in the t6sP of EHEC EDL933. [file spectrum.03548-25-s0006.tif]

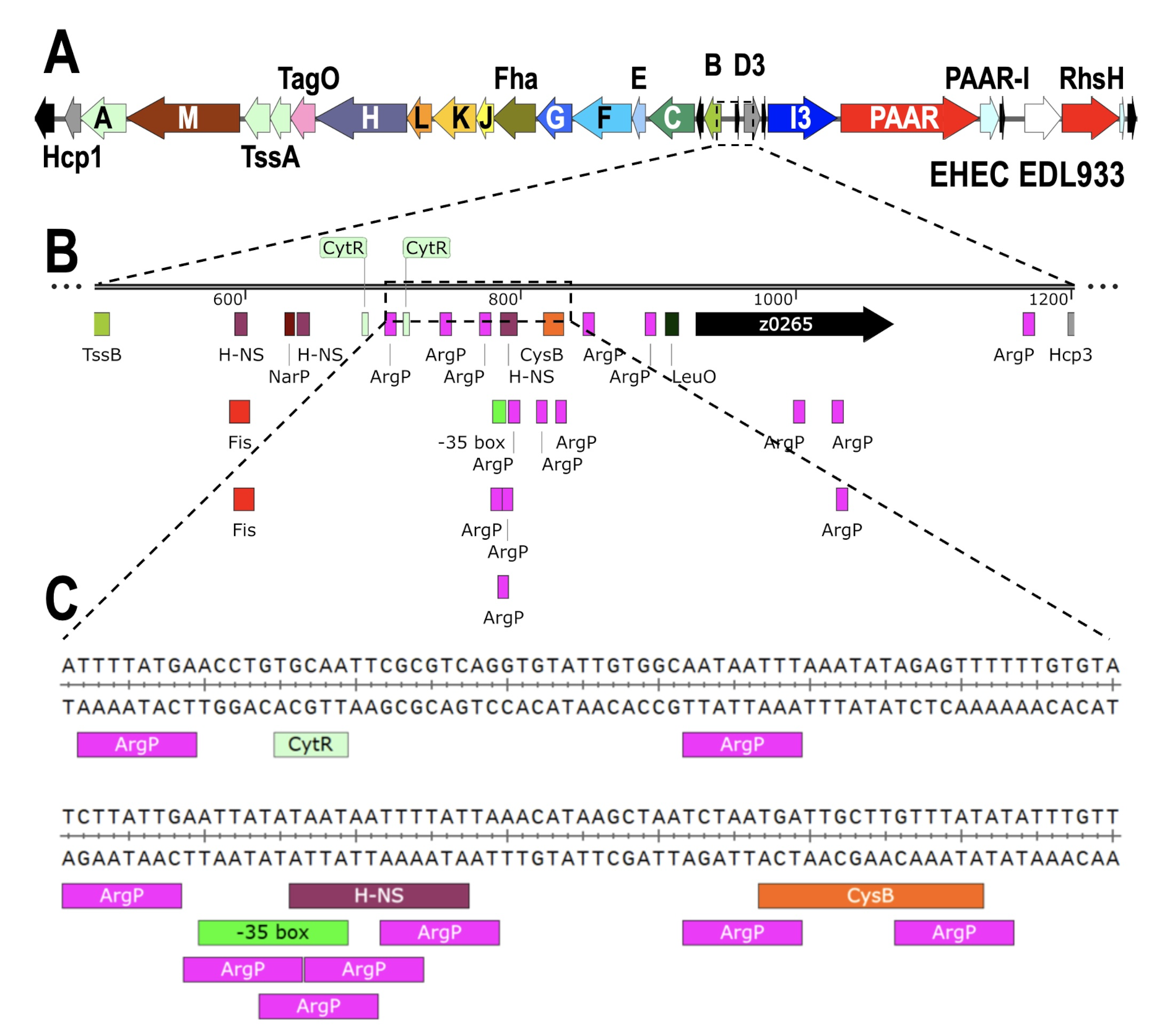

Supplement: Fig. S5 — Deletion of the hns gene negatively impacts growth and only modestly affects expression of T6SS-related proteins. [file spectrum.03548-25-s0007.tif]

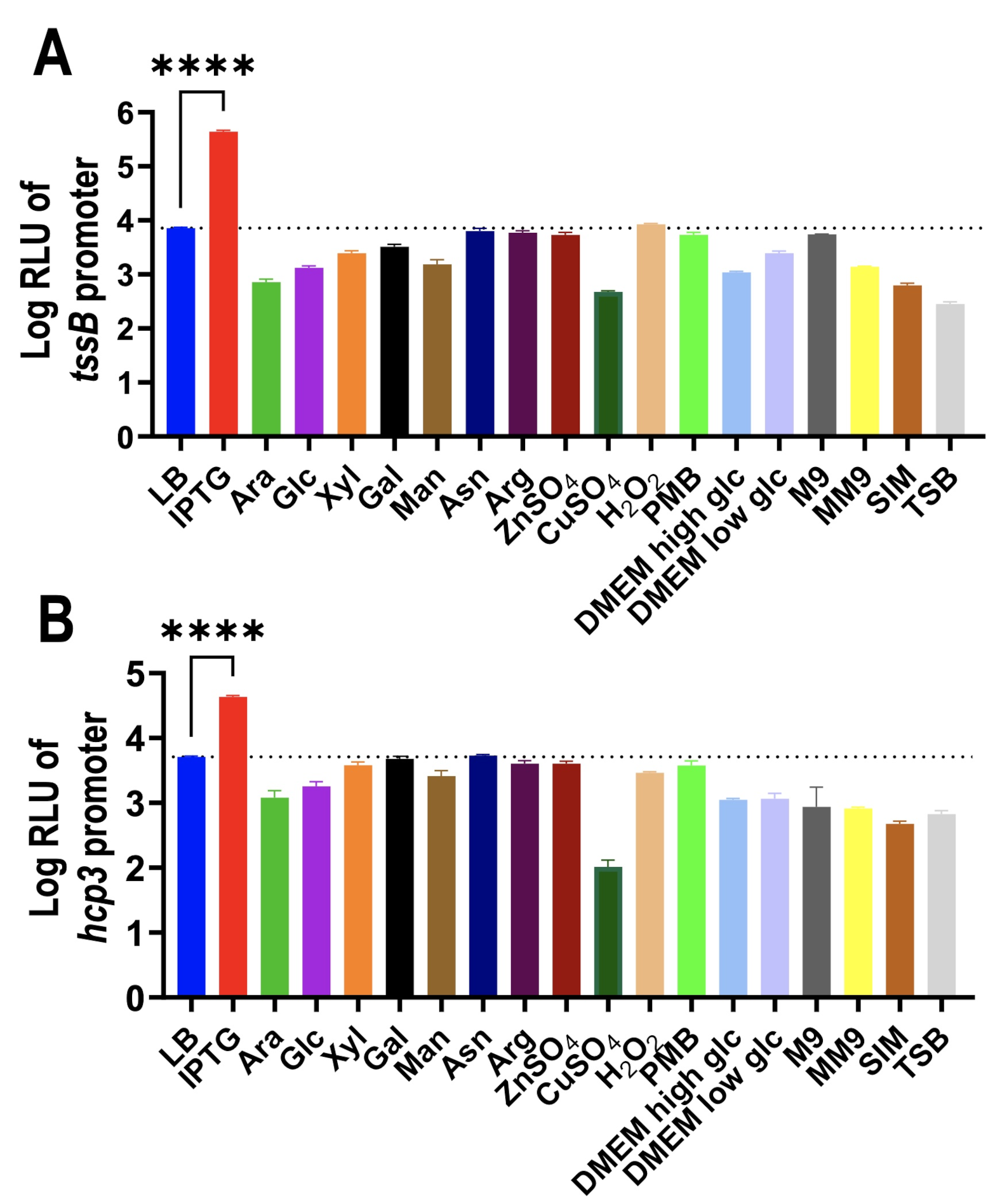

Supplement: Fig. S6 — Analysis of t6sP in different media using ilux. [file spectrum.03548-25-s0008.tif]
